# Temporal and spatial dynamics of Net Primary Productivity and prediction of wetland carbon sequestration potential on the Tibetan Plateau

**DOI:** 10.7717/peerj.20758

**Published:** 2026-02-04

**Authors:** Liang Cao, Shi Dong, Yuyan Wang, Xingran Li, Yonghua Zhao, Danni Ma, Zhuoma Pubu, Hongmei Ma, Wei Li, Pengxi Cao

**Affiliations:** 1 Key Laboratory of Biodiversity and Environment on the Qinghai-Tibetan Plateau, Ministry of Education, School of Ecology and Environment, Xizang University, Lhasa, China; 2 Nagqu Mitika, Alpine Wetland Ecosystem Observation and Research Station of Tibet Autonomous Region, Xizang University, Nagqu, China; 3 Collaborative Innovation Center for Ecological Civilization of the Qinghai-Tibetan Plateau, Xizang University, Lhasa, China; 4 Institute of Plateau Biology of Xizang Autonomous Region, Lhasa, China

**Keywords:** Net Primary Productivity, Carbon sequestration in wetlands, Normalized Difference Vegetation Index, Land-use change, Future projections

## Abstract

**Background:**

Investigating the carbon sequestration potential of wetlands and the dynamics of net primary productivity (NPP) on the Tibetan Plateau of China enhances understanding of their contributions to global carbon emission reduction and their role in maintaining biodiversity and ecosystem stability. Vegetation NPP is a key indicator of carbon sequestration; however, existing research has largely focused on historical dynamics, with limited studies projecting future trends. This gap impedes proactive conservation and climate mitigation strategies.

**Methods:**

Here, we predicted the spatial distribution of normalized difference vegetation index (NDVI) on the Tibetan Plateau for 2025–2030, employing a backpropagation neural network and Kriging interpolation fitting. We estimated spatial and temporal dynamics of NPP and wetland carbon sequestration potentials during the same period using the Carnegie-Ames-Stanford Approach model. Furthermore, we investigated the effects of land use and climate on NPP.

**Results:**

Key findings were: (1) NDVI distribution was higher in the southeast than in the northwest, with temperature influencing its value. (2) Spatial distribution of NPP on the Tibetan Plateau exhibits a typical landscape pattern of “patch-corridor-matrix.” The maximum NPP of vegetation was 1,112.82 gC ⋅ m^−2^ ⋅ a^−1^ for 2025–2030. Projections of NPP for 2025–2030 suggest an increase of approximately 50% relative to current levels by 2045, indicating a substantial enhancement of the carbon sink potential over the coming two decades. (3) Carbon sequestration potential of wetlands on the Plateau ranges from 0 to 100 gC ⋅ m^−2^ ⋅ a^−1^, with high carbon sink potential concentrated near Palong Tsangpo, the largest tributary of the Yarlung Tsangpo River. (4) Woodland NPP has the highest mean value and rate of change. Furthermore, analysis of 2025 land use data shows that forestland and grassland are the dominant land cover types in the Yunnan, Sichuan, and Southeastern Xizang Sections of the Qinghai-Tibet Plateau. Their high proportions correspond significantly to high regional NDVI values, indicating the spatial heterogeneity of NDVI distribution is driven by land cover changes rather than directional factors. (5) Correlation analysis indicated strong positive correlations between precipitation and solar radiation with NPP. NPP does not increase or decrease with increasing temperature; instead, it tends to increase within suitable temperature ranges.

## Introduction

Global climate change, coupled with China’s “dual carbon” strategy—a national initiative formally proposed in 2020, aiming for peak carbon dioxide emissions by 2030 and carbon neutrality by 2060 (Zhang, Zheng & Ling, 2022)—have highlighted the growing need for research on vegetation net primary productivity (NPP) and wetland carbon sequestration. Vegetation NPP serves as an important indicator of carbon sequestration potential, while the carbon sequestration capacity is an essential measure of ecosystem health and stability. Investigating vegetation NPP enhances our understanding of the adaptive capacity of an ecosystem to climate change and its stability in the face of disturbances, thereby providing theoretical support for ecosystem protection (Wu et al., 2024). The Tibetan Plateau in China, referred to as the “Roof of the World,” “Third Pole,” and “Water Tower of Asia,” occupies a key position in the global ecological framework. Its extensive grasslands, forests, and wetlands possess considerable potential for carbon sequestration (Zhang et al., 2023). However, research predicting the NPP and wetland carbon sequestration potential of the Tibetan Plateau for the critical period of 2025–2030 remains limited. Accurately predicting the spatial and temporal dynamics of NPP and the carbon sequestration potential of wetlands during this period can clarify carbon sink dynamics, guide vegetation protection and restoration in fragile ecological zones, and contribute to ecosystem balance and stability (Zhang et al., 2025a). This enhances our understanding of the carbon cycle mechanisms in plateau ecosystems and provides a scientific basis for regional ecological protection policies and climate change strategies.

Wetlands, as unique and ecologically important ecosystems, play a pivotal role in the global carbon cycle. Covering only 5–8% of Earth’s terrestrial surface, they store approximately one-third of the planet’s terrestrial soil organic carbon (SOC) (Bridgham et al., 2013; Saunois et al., 2020). The global decline of wetlands underscores the growing importance of wetland conservation (Qu et al., 2024). Research has indicated that the global wetland area has declined by 50% since 1900 and may have declined by 87% since 1700 (Davidson, 2014). The alarming loss of wetlands has led to 25% of global inland wetland-dependent species being listed as threatened (Qu et al., 2024). The wetlands of the Tibetan Plateau are an essential component of the global carbon cycle. The alpine grasslands on the Qinghai-Tibetan Plateau are estimated to store approximately 0.80 and 7.50 Pg C in their vegetation and the topsoil (0–30 cm), respectively (Han et al., 2022). Conserving the carbon stored in these wetlands contributes to the mitigation of atmospheric CO_2_ concentrations. In contrast, wetland degradation may trigger a vicious cycle: increased carbon release accelerates global warming, which further exacerbates wetland loss, ultimately affecting global climate patterns. Therefore, numerous scholars have contributed to research on carbon sequestration prediction in wetlands. For example, the spatial and temporal dynamics and factors influencing wetland carbon sinks in Dongting Lake have been explored using multiple analytical models (Guo et al., 2025). Ouyang, Maher & Santos (2024) employed a random forest model incorporating precipitation, evapotranspiration, and temperature as covariates to investigate the effects of climate change on global carbon export from groundwater in tidal wetlands. The coefficient of determination (*R*^2^) values of the model in mangrove forests and tidal marshes were 0.6 and 0.96, respectively, indicating strong predictive accuracy. Their findings suggest that environmental changes may weaken the capacity of tidal wetlands to act as carbon sinks and mitigate ocean acidification. Yang et al. (2025) focused on the Qaidam peatlands in the central Qinghai-Tibet Plateau, validating proxy indicators (*e.g.*, peat carbon isotopes), establishing an accurate radiocarbon chronology, and calibrating against global datasets to ensure accuracy. The findings reveal that since approximately 1,000 cal yr B.P., climate warming and drying have led to decreased groundwater levels, thereby reducing carbon and nitrogen accumulation rates. However, the peatlands in this region remain global hotspots for carbon and nitrogen sequestration. Additionally, Yang et al. (2025) included soil moisture content, temperature, and precipitation served as covariates in the study to analyze the relationship between carbon-nitrogen sequestration and environmental factors. Ruoergai alpine wetland data were extracted for 1985–2020. The SOC content was predicted using a random forest model, yielding an *R*^2^ value of 0.89 and demonstrating statistical significance (*p* < 0.001) for the regression model used to harmonize SOC data across various soil layers. The Ruoergai wetland experienced 378.71 km^2^ degradation between 1985 and 2020, while its total SOC storage increased from 2.03 to 2.21 Pg. Additionally, climate factors were incorporated as covariates in the model (Zhang, Cheng & Wu, 2023). In their study, Zhang, Cheng & Wu (2023) used air temperature as a covariate to account for the influence of ambient atmospheric temperature on NDVI, thereby preventing interference with the predictive effect of the core soil-moisture factor.

The maturity of 3S technologies, including remote sensing (RS), geographic information systems, and global positioning systems, has rendered remotely sensed observations a valuable tool for NPP estimation (Chen et al., 2022). The following serve as representative examples: (1) Empirical models: the Miami (Xu et al., 2024b) and Thornthwaite Memorial (Xu et al., 2024a) models; (2) process models: Carnegie Ames Stanford Approach (CASA) (Lv et al., 2024) and Biome-BioGeochemical Cycles (Fang et al., 2024) models; and (3) light energy utilization models: Global Production Efficiency Model (Ruimy et al., 1999) and Vegetation Photosynthesis (Gong et al., 2024) models. Empirical models are simple and efficient; however, they lack a robust mechanistic basis and have limited applicability. Conversely, process-based models are underpinned by a strong mechanistic basis and offer broad applicability; however, they are complex and demand high data and computational resources. Light energy utilization excels in simulating light energy conversion and balance efficiency with remote sensing compatibility; however, they tend to simplify numerous other ecological processes. Among them, the CASA model is an ecological model used to estimate the NPP of terrestrial ecosystems; it is based on RS and comprehensively considers the effects of climatic factors, including temperature, precipitation, and radiation, and soil factors on vegetation growth. The role of the natural environment in shaping ecosystem dynamics can be more comprehensively reflected through this model (Qi, Zhang & Zhang, 2023). The application of the CASA model to estimate vegetation NPP has been widely recognized by scholars worldwide, and its accuracy has been proven to meet the requirements of research related to vegetation dynamics (Liu, 2024).

One of the most important indicators used in constructing the CASA model is the NDVI. As the CASA model has been widely used to estimate NPP on the Tibetan Plateau, an area sensitive to climate change and critical for regional carbon cycling, the accuracy of future NDVI datasets has been shown to directly determine the reliability of CASA-based NPP spatio-temporal change predictions. However, the harsh environmental conditions of the Tibetan Plateau and complex vegetation–climate interactions have presented considerable challenges to obtaining accurate future NDVI, making its reliable prediction particularly important and necessary for improving the credibility of subsequent NPP assessments. Numerous scholars have conducted in-depth studies on prediction models across various fields: (1) Time-series forecasting models: moving average models (Xu, 2021), exponential smoothing method (Smyl et al., 2025), and ARIMA model (Wang et al., 2025); (2) regression analysis prediction models: linear (Lee, Lee & Choi, 2025), nonlinear (Liu, 2011), and multiple regression (King, Kim & Yune, 2024); (3) machine learning prediction models: decision trees (Jia et al., 2025), random forests (Li et al., 2025), and support vector machines (Hou et al., 2025); and (4) deep learning prediction models: artificial neural networks (Wang et al., 2024), recurrent neural networks (Wen, Weng & Xie, 2024), convolutional neural networks (Martínez et al., 2024), and gray prediction models (Jiang, Zhang & Huang, 2025). A backpropagation (BP) neural network, a multilayer feedforward neural network trained according to the error BP algorithm, has been shown to handle highly complex nonlinear relationships between inputs and outputs. Trained BP neural networks have demonstrated strong predictive capabilities. In this study, a BP neural network was fitted with Kriging interpolation to predict the spatial variability of NDVI and improve its prediction accuracy. Therefore, this study proposed a new conceptual framework for the CASA model to predict future spatial and temporal changes in NPP.

The Tibetan Plateau occupies a key position in the terrestrial ecosystem. This entity exhibits great potential for carbon sequestration, serves as a core component of carbon sources and sinks within terrestrial ecosystems, and plays an indispensable role in the global carbon balance (Qi et al., 2012). However, research on the future time series of NPP and carbon sequestration potential of wetlands is lacking. Based on this, we propose the following hypotheses: (1) Studies have shown that the overall trend of NPP in grassland on the Tibetan Plateau between 1978 and 2020 decreases from southeast to northwest (Xu & Zhang, 2024); therefore, we propose that the spatial distribution of NPP on the Plateau follows a gradient of high values in the southeast and low values in the northwest; (2) based on previous studies on the effects of climatic factors on NPP, we hypothesize that precipitation and solar radiation strongly influence NPP (Tang, Peng & Peng, 2025); and (3) it is suggested that carbon sequestration rate by vegetation on the Tibetan Plateau will peak between 2025 and 2035, leading to a fluctuating upward trend in carbon sink potential from 2025 to 2030 (Cai et al., 2025).

## Materials and Methods

### Study area

**Figure 1:**
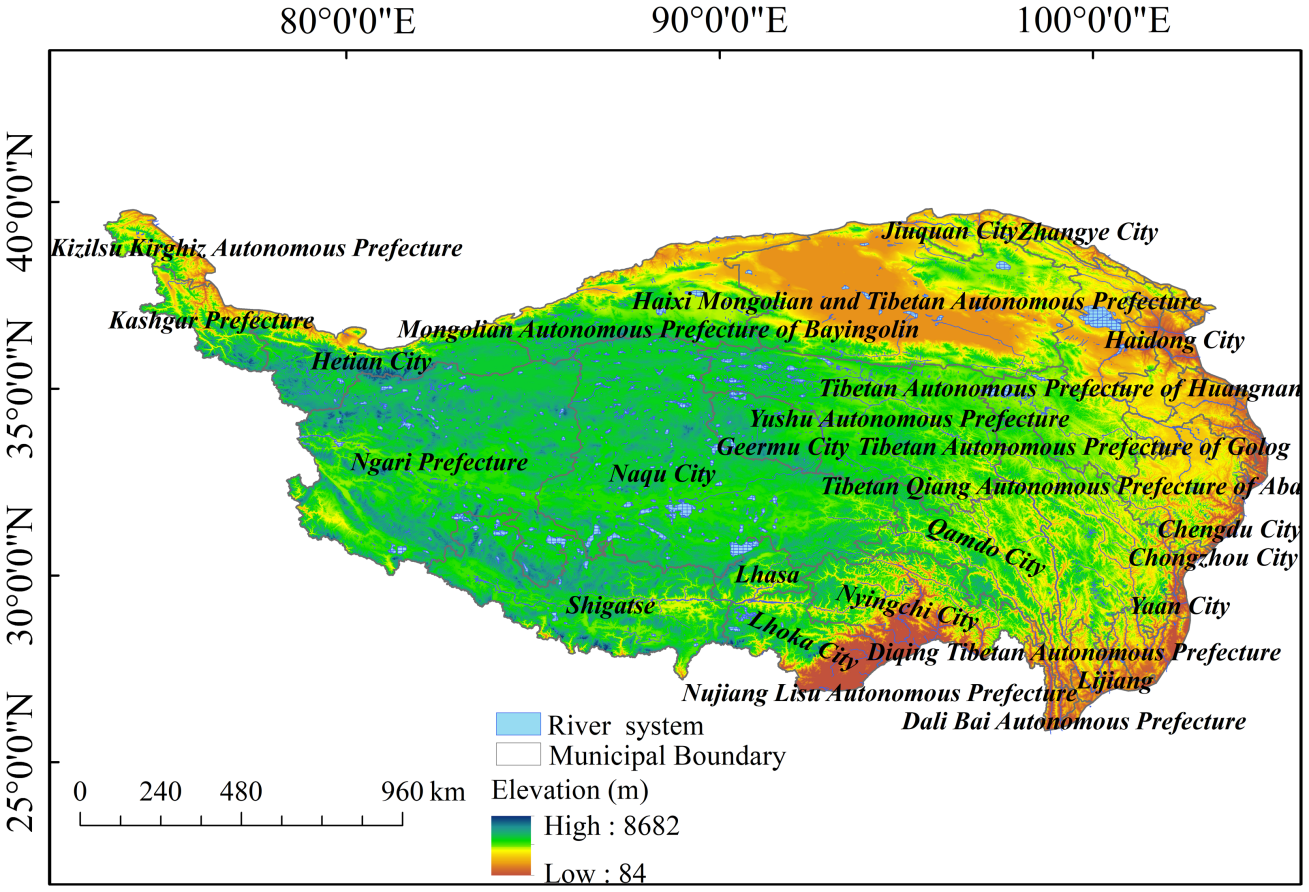
Elevation map of the Tibetan Plateau, China.

The Tibetan Plateau, China, is situated between latitudes 26°00′–39°47′N and longitudes 73°19′–104°47′E, encompassing a total area of approximately 2.5 million km^2^. Located within the territory of China, the Qinghai-Tibetan Plateau, the highest plateau globally, has an average elevation of >4,000 m above sea level and includes parts of Xizang, Qinghai, Xinjiang, Gansu, Sichuan, and Yunnan (Fig. 1). The region is characterized by a complex network of mountain ranges, dominated by the Himalayas and Kunlun Mountains. Alongside mountain ranges, vast plateau surfaces and basins are scattered throughout, forming a diverse topography with varying elevations and depressions. The northwest region features high terrain, with the highest point at 8,682 m, while the southeast region exhibits lower terrain, with the lowest point at 84 m. This area includes glaciers and snow-covered mountains at high altitudes and various landforms at low altitudes, such as deep alpine valleys, open plateau meadows, and vast Gobi deserts, all of which vary with altitude (Zhang, Li & Zheng, 2002).

The Tibetan Plateau exhibits a low annual average temperature, decreasing from 20 °C in the southeast to below −6 °C in the northwest. Day and night temperatures vary considerably, primarily due to the thin air on the plateau, limited atmospheric insulation, strong solar radiation during the daytime, rapid ground heating, and subsequent rapid radiating heat loss at night, resulting in a considerable temperature decrease. The temperature decreases with increasing altitude, with an approximate decrease of 0.6 °C for every 100 m of elevation, resulting in a unique alpine climate (Zhao et al., 2024). The spatial distribution of precipitation is uneven, particularly in the southeast, which is influenced by the southwest monsoon. This phenomenon results in the influx of warm and humid air currents that bring abundant precipitation. Annual precipitation can reach >800 mm in certain regions, while the northwest, being deep inland, experiences minimal influence from the monsoon and exhibits an arid climate with <200 mm of annual precipitation. The seasonal distribution of precipitation is uneven, primarily occurring in the summer months when water vapor from summer winds generates rainfall on the plateau, whereas winter and spring months experience limited precipitation. The evolution of alpine grassland vegetation on the Tibetan Plateau over the past 40 years has exhibited considerable spatial non-uniformity. The observed differences are primarily governed by geographic variation, which is largely dominated by climatic factors that substantially impact carbon sequestration potential (Wang et al., 2023a).

### Data sources and processing

In this study, we utilized the scientific database “Bias-corrected CMIP6 global dataset for dynamical downscaling of the Earth’s historical and future climate (1979–2100)” (https://www.scidb.cn/en/detail?dataSetId=791587189614968832) along with data from the China Tibetan Plateau Science Data Center (https://data.tpdc.ac.cn/). The data encompassed climatic variables (precipitation, temperature, relative humidity, and solar radiation), physical parameters (soil temperature and soil moisture), and NDVI data. The China Land Cover Dataset was derived from the high-resolution land cover dataset developed by the team of Prof. Yang Jie and Prof. Huang Xin at Wuhan University and dataset published in *Nature*’s subjournal *Scientific Data* by the team of Associate Prof. Wu Xudong from the School of Soil and Water Conservation at Beijing Forestry University, featuring a specific temporal and spatial resolution (Table 1).

**Table 1:** Data sources and descriptions.

Data	Year	Resolution/ scale	Source
Monthly precipitation	2001–2010; 2025–2030	1 km	Zhao, Wang & Ma (2024); Peng (2022)
Average monthly air temperature	2001–2010; 2025–2030	1.25°	Xu et al. (2024c)
Average monthly relative humidity	2001–2010	1.25°	Xu et al. (2024c)
Soil humidity	2001–2010	1.25°	Xu et al. (2024c)
Soil temperature	2001–2010	1.25°	Xu et al. (2024c)
Surface solar radiation	2001–2010; 2025–2030	0.1°	Tang, He & Hong (2023)
Land use data	2023; 2020 and 2030	30 m; 1 km	Yang & Huang (2024); Zhang, Cheng & Wu (2023)
NDVI	2001–2010; 2020–2024	0.25°; 0.25 km	Zhong & Wang (2023); Gao et al. (2023)

Elevation data were sourced from the second generation of Advanced Spaceborne Thermal Emission and Reflection Radiometer Global Digital Elevation Model (DEM), a joint collaboration between NASA (USA) and the Ministry of Economy, Trade, and Industry (Japan), featuring a spatial resolution of 30 m (NASA/METI/AIST/Japan Spacesystems & US/Japan ASTER Science Team Team, 2018). Data were downloaded from the Geospatial Data Cloud (https://www.gscloud.cn/). Based on ArcGIS, raw data pre-processing facilitated the acquisition of DEM data for the Tibetan Plateau region.

**Figure 2:**
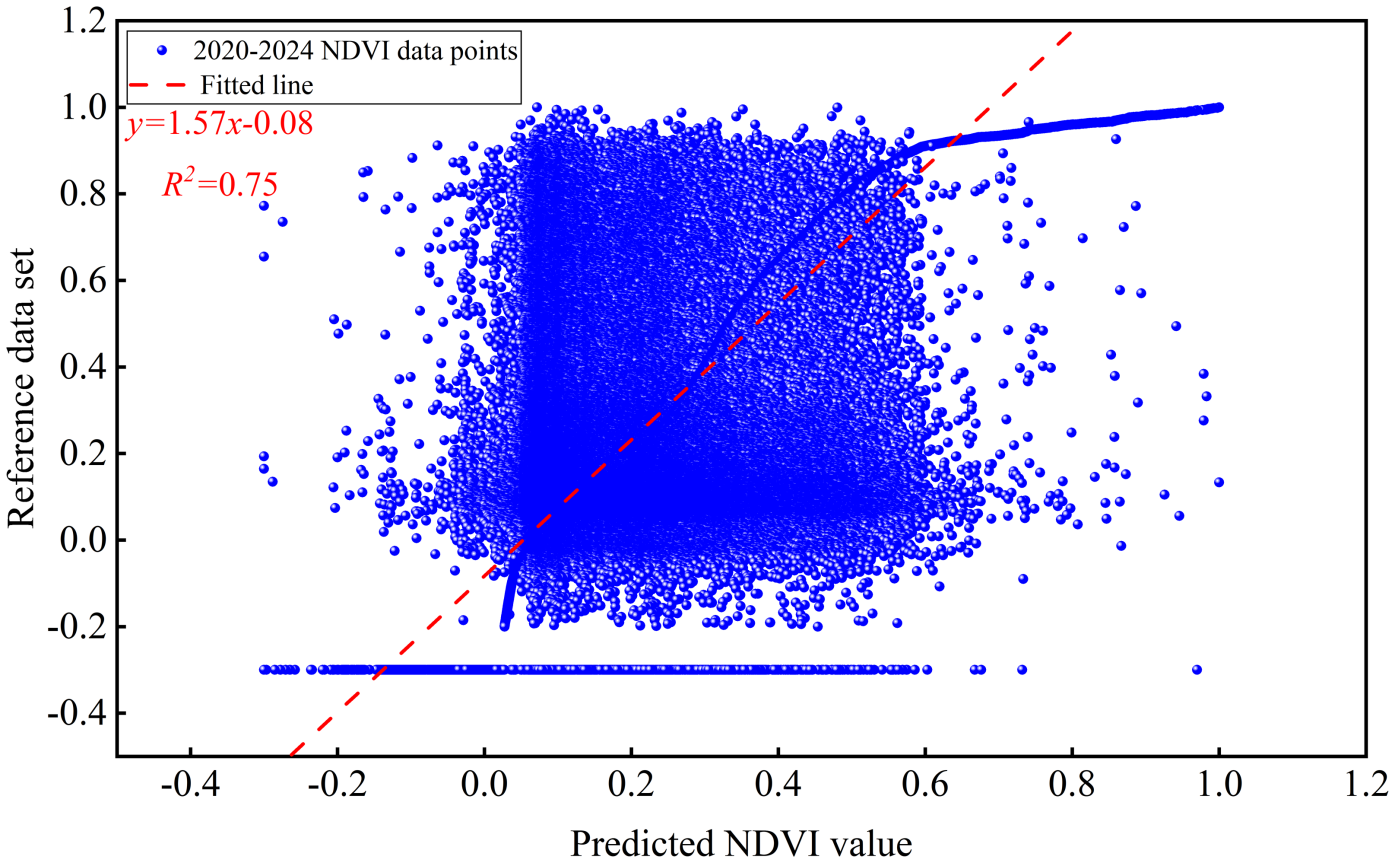
Linear fitting comparison between predicted values and measured dataset of NDVI.

**Figure 3:**
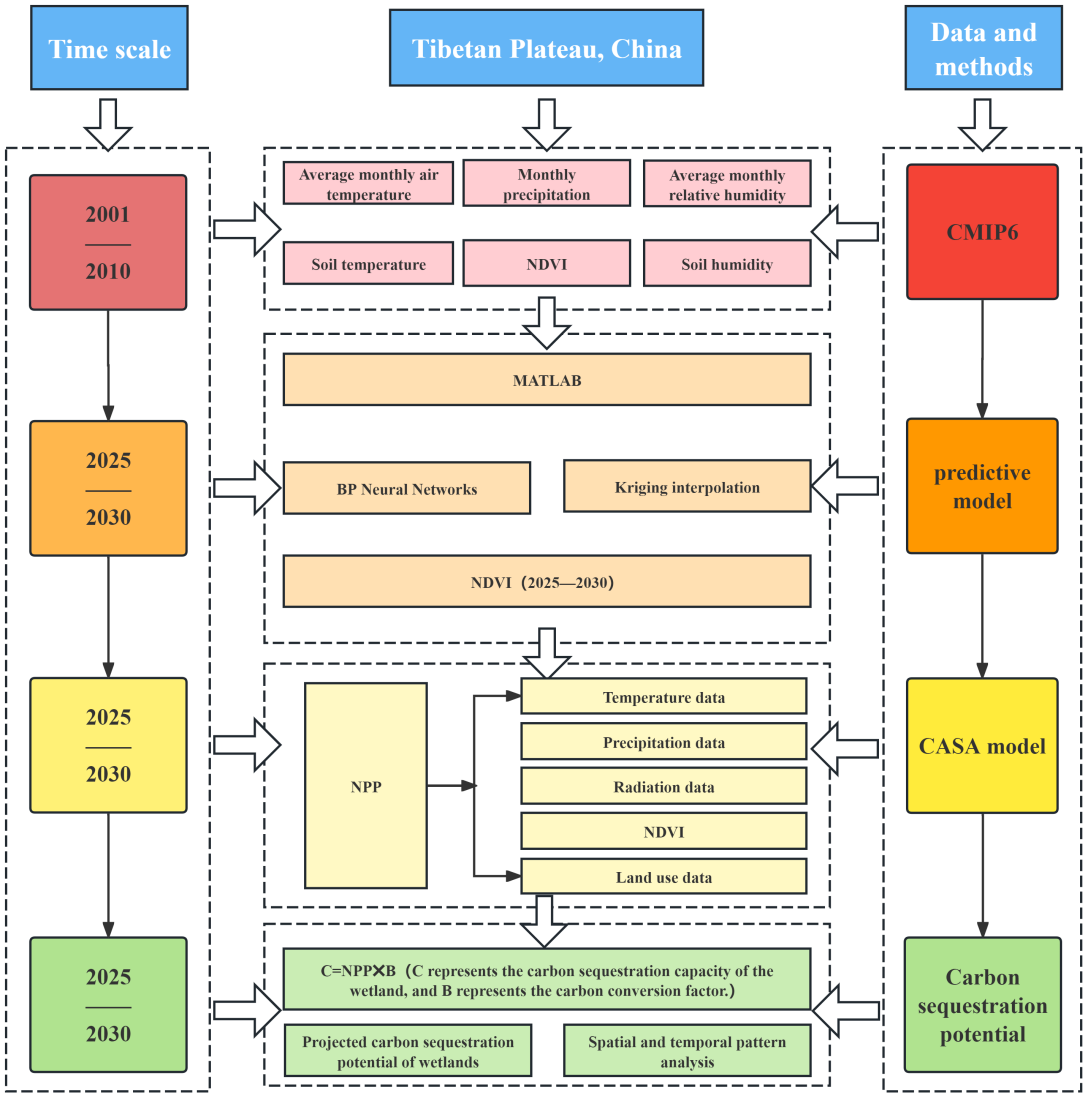
Flowchart of research methodology.

All climate, physical, NDVI, and land-use data were uniformly resampled to an image pixel size of 0.011305581° (approximately 1,255 m, based on the average latitude of the study area) and reprojected to a consistent coordinate system. A BP neural network prediction model was developed using MATLAB, and the values of each pixel were extracted. Input layers consisted of monthly average temperature, precipitation, relative humidity, soil temperature, and soil humidity between 2001 and 2010. The output data represent NDVI data from 2001 to 2010. The NDVI data generated by the BP neural network model were produced as discrete grid points. However, for the subsequent NPP estimation using the CASA model in this study, spatially continuous NDVI data were required as the core input, a criterion not satisfied by the discrete-point format. The Kriging interpolation method was employed to process the discrete NDVI grid points, thereby addressing this discrepancy. As a technique for the unbiased optimal estimation of attribute values at unknown locations, Kriging interpolation operates by analyzing the distribution of geographic variables and structure of their variograms. The interpolation process fully considers the spatial positional relationships between unknown and all known sample points while also leveraging the spatial structural characteristics inherent in the data of each sample point. This approach ensures the generation of continuous NDVI data suitable for subsequent NPP calculations (Yang et al., 2023). It is worth noting that the NDVI data from 2001 to 2010 was selected as the training set due to its completeness with no missing values. To verify the accuracy of predicting data from 2025 to 2030, the NDVI data from 2020 to 2024 was used as the validation set (the data was sourced from the China regional 250 m normalized difference vegetation index data set (Gao et al., 2023)). With a validation *R*^2^ of 0.75 (Fig. 2), the model demonstrates good fitting performance and can support the prediction of NDVI data from 2025 to 2030. The NPP dataset for 2025–2030 was calculated using CASA modeling (Fig. 3).

### Methods

#### BP neural network prediction model

In 1986, a research team led by Rumelhart and McClelland proposed the BP neural network (Shavlik & Towell, 1989). This structure is a multilayer feedforward network trained using an error backpropagation algorithm, representing one of the most widely used neural network models currently (Chai & Kun, 2021). BP neural networks possess the advantages of approximating arbitrary nonlinear mapping relations, enhanced generalization ability, improved fault tolerance, and simple implementation (Wang, 2015).

Core formulas

(1) Hidden layer output:

(2.1) \begin{eqnarray*}{h}_{j}=f \left( \sum _{i=1}^{n}{w}_{ij}{x}_{i}+{b}_{j} \right) .\end{eqnarray*}

(2) Output layer output:

(2.2) \begin{eqnarray*}{y}_{k}=g \left( \sum _{j=1}^{m}{w}_{jk}{h}_{j}+{b}_{k} \right) .\end{eqnarray*}

Where:

*x*_*i*_: Input variables; *h*_*j*_: Output of the j-th hidden layer neuron; *y*_*k*_: Model predicted value.

*w*_*ij*_: Weight between the i-th input and j-th hidden neuron; *w*_*jk*_: Weight between the j-th hidden neuron and k-th output neuron; *b*_*j*_: Bias of the j-th hidden neuron; *b*_*k*_: Bias of the k-th output neuron.

*f*(⋅): Hidden layer activation function (commonly Sigmoid function: $f(x)= \frac{1}{1+{e}^{-x}} $; *g*(⋅): Output layer activation function (linear function for regression tasks).

#### Model accuracy verification

 (1) Correlation coefficient (R)

For two variables, *x* and*y*, the sample correlation coefficient *R* is defined as follows:

(2.3) \begin{eqnarray*}R= \frac{\sum _{i=1}^{n}({x}_{i}-x^{-})({y}_{i}-y^{-})}{\sqrt{\sum _{i=1}^{n}({x}_{i}-x^{-})^{2}}\sqrt{\sum _{i=1}^{n}({y}_{i}-y^{-})^{2}}} \end{eqnarray*}

where *n* is the sample size; *x*_*i*_ and *y*_*i*_ are the *i*th observation of variables *x* and *y*, respectively; and $x^{-}$ and $y^{-}$ are the sample means of *x* and *y*, respectively.

(2) Model robustness validation (Monte Carlo simulation)

A Monte Carlo perturbation simulation was conducted to assess the robustness of the neural network model in the presence of input uncertainties. The procedure is summarized as follows:

(a) Input perturbation

The original input matrix was represented as follows:

(2.4) \begin{eqnarray*}p= \left\{ {p}_{ij} \right\} ,i=1,\ldots ,m,j=1,\ldots ,n\end{eqnarray*}

where *m* is the number of features, and *n* is the number of samples.

At the *k-* th simulation, each input element can be perturbed as follows:

(2.5) \begin{eqnarray*}{p}_{ij}^{ \left( k \right) }={p}_{ij}\cdot (1+\rho \cdot (2{r}_{ij}-1))\end{eqnarray*}

where *ρ* = 0.02 is the perturbation ratio (±2%), and *r*_*ij*_ ∼ *U*(0,1) is a random number uniformly distributed in [0,1].

(b) Model prediction

The perturbed input ${p}^{ \left( k \right) }$ was normalized and then fed into the trained neural network model *f*():

(2.6) \begin{eqnarray*}{y}^{ \left( k \right) }=f({p}^{ \left( k \right) };\theta ),k=1,\ldots ,N\end{eqnarray*}

where *θ* is the fixed network parameter. The number of simulations was set to *N* = 300.

(c) Statistical analysis

The outputs $ \left\{ {y}^{ \left( 1 \right) },\ldots ,{y}^{ \left( N \right) } \right\} $ were aggregated for statistical evaluation:

Mean prediction:

(2.7) \begin{eqnarray*}y^{-}= \frac{1}{N} \sum _{k=1}^{N}{y}^{ \left( k \right) }.\end{eqnarray*}

Standard deviation (uncertainty):

(2.8) \begin{eqnarray*}{\sigma }_{y}=\sqrt{ \frac{\sum _{k=1}^{N}({y}^{ \left( k \right) }-y^{-})^{2}}{N-1} }.\end{eqnarray*}

Mean squared error (MSE) distribution:

(2.9) \begin{eqnarray*}MS{E}^{ \left( k \right) }={ \frac{\sum _{j=1}^{n}({y}_{j}^{ \left( k \right) }-{\hat {y}}_{j})}{n} }^{2}\end{eqnarray*}

By calculating the mean and standard deviation of $MS{E}^{ \left( k \right) }$, the robustness of the model under input perturbations can be quantitatively assessed. Moreover, plotting the prediction mean alongside the confidence band (mean ±1 standard deviation) provides a visual representation of the variability in model outputs.

(3) Normalized root mean square error (NRMSE)

(2.10) \begin{eqnarray*}NRMSE= \frac{RMSE}{{y}_{\mathrm{max}}-{y}_{\mathrm{min}}} \times 100\%\end{eqnarray*}

(2.11) \begin{eqnarray*}RMSE=\sqrt{ \frac{\sum _{i=1}^{n}(\widehat{{y}_{i}}-{y}_{i})^{2}}{n} }\end{eqnarray*}

where *n* is the number of samples; *y*_*i*_ is the *i*th observed (true) value; ${\hat {y}}_{i}$ is the *i*th predicted value; *y*_max_ is the maximum of the observed values; and *y*_min_ is the minimum of the observed values.

#### Kriging interpolation

In this study, the ordinary Kriging interpolation method was employed, while the spherical function was selected as the variational function for the interpolation calculation. In contrast to inverse distance weighting and spline interpolation, Kriging interpolation is based on the variational function model. This approach effectively utilizes the spatial autocorrelation information of the data and enables the optimal unbiased estimation of unknown points (Tang & Yang, 2021).

Assuming that the target interpolation point is *p*_0_, number of sample points is known to be *p*_1_, *p*_2_, ⋅⋅⋅, *p*_*m*_, and observation corresponding to the sample point is *f*(*p*_1_), *f*(*p*_2_), ⋅⋅⋅, *f*(*p*_*m*_), the estimate $\tilde {f}({p}_{0})$ at *p*_0_ obtained through Kriging interpolation is calculated as follows (Tang & Yang, 2021):

(2.12) \begin{eqnarray*}\tilde {f}({p}_{0})=\sum _{k=1}^{m}{w}_{k}f({p}_{k})\end{eqnarray*}

where *w*_*k*_ is the weight coefficient and satisfies ${\mathop{\sum }\nolimits }_{k=1}^{m}{w}_{k}=1$.

#### CASA modeling

The CASA model was developed by a team of researchers from the Carnegie Institution, Stanford University, and NASA’s Ames Research Center (Potter et al., 1993). Precipitation, temperature, radiation data, and Normalized Vegetation Index (NDVI) data for the same period were obtained for the span of 2025–2030 for this scientific work. The NDVI data were predicted using a BP neural network. Based on the CASA model, the net primary productivity (NPP) of the target study area was simulated so as to analyze the dynamics of the ecosystem productivity in the area and reveal its evolution in a long time series.

In the CASA model, the calculation of net primary productivity (NPP) of vegetation follows this approach: the value of net primary productivity of vegetation for a given image element *x* at moment *t* is equal to the product of the photosynthetically active radiation absorbed by that image element *x* at moment *t* and the actual light energy utilization of that image element *x* at moment *t*. It is expressed in a mathematical formula as:

(2.13) \begin{eqnarray*}NPP(x,t)& =APAR(x,t)\times (x,t)\end{eqnarray*}

(2.14) \begin{eqnarray*}APAR(x,t)& =SOL(x,t)\times FPAR(x,t)\times 0.5\end{eqnarray*}

(2.15) \begin{eqnarray*}FPAR(x,t)& =0.5\times (FPA{R}_{NDVI(x,t)}-FPA{R}_{SRi,\min \nolimits })\end{eqnarray*}

(2.16) \begin{eqnarray*}FPA{R}_{NDVI(x,t)}=(NDVI(x,t)-NDV{I}_{i,\min \nolimits })\times \frac{FPA{R}_{\mathrm{max}}-FPA{R}_{\mathrm{min}}}{NDV{I}_{i,\max \nolimits }-NDV{I}_{i,\min \nolimits }} +FPA{R}_{\mathrm{min}}\end{eqnarray*}

(2.17) \begin{eqnarray*}FPA{R}_{SR(x,t)}& =(SR(x,t)-S{R}_{i,\min \nolimits })\times \frac{FPA{R}_{\mathrm{max}}-FPA{R}_{\mathrm{min}}}{S{R}_{i,\max \nolimits }-S{R}_{i,\min \nolimits }} +FPA{R}_{\mathrm{min}}\end{eqnarray*}

(2.18) \begin{eqnarray*}SR(x,t)& = \frac{1+NDVI(x,t)}{1-NDVI(x,t)} .\end{eqnarray*}

In this equation, *NPP*(*x*, *t*) represents the net primary productivity of the vegetation of pixel *x* at the moment of *t*, which has the unit of gC⋅m^−2^⋅a^−1^; *APAR*(*x*, *t*) is the photosynthetically active radiation absorbed by the pixel at the moment, which has the unit of MJ⋅m^−2^⋅a^−1^; *FPAR*_*NDVI*(*x*,*t*)_ is the proportion of incident photosynthetically active radiation absorbed by the vegetation layer calculated from the NDVI; *SOL*(*x*, *t*) represents the total solar radiation at spatial location *x* and time *t*; *FPAR*(*x*, *t*) generally refers to the proportion of photosynthetically active radiation of vegetation; *SR*(*x*, *t*) is the ratio vegetation index of image x in month t; *FPAR*_*SR*(*x*,*t*)_ is the proportion of incident photosynthetically active radiation absorbed by the vegetation layer calculated from the ratio vegetation index; *NDVI*(*x*, *t*) is the NDVI of image *x* in month *t*; *NDVI*_*i*,min_ and *NDVI*_*i*,max_ are the minimum and maximum values of NDVI for the ith planting type, respectively; *SR*_*i*,min_ and *SR*_*i*,max_ are the minimum and maximum values of *SR* for the *i*th planting type, which are calculated from *NDVI*_*i*,min_ and *NDVI*_*i*,max_ for the corresponding *i*th planting type, respectively; *FPAR*_max_ and *FPAR*_min_ are the percentages of incident photosynthetically active radiation absorbed by the vegetation layer, respectively. *FPAR*_max_ and *FPAR*_min_ are the maximum and minimum proportions of incident photosynthetically active radiation absorbed by the vegetation layer, which are 0.95 and 0.001, respectively; and *ɛ*(*x*, *t*) while represents the actual light energy utilization of the pixel *x* at the moment of *t*, which has the unit of gC⋅*Mj*^−1^.

(2.19) \begin{eqnarray*}C(x,t)=NPP\times B.\end{eqnarray*}

C represents the carbon sequestration potential of the wetland, NPP is the net primary productivity of the wetland vegetation, and B is the carbon conversion factor of the wetland, which takes the value of 0.44 g (Shen et al., 2022).

#### Transfer matrix

The land use transfer matrix is typically employed to characterize the conversion relationships among various land use types between the initial and final time points within a specific time interval (Zhang et al., 2017).

Core formulas

(2.20) \begin{eqnarray*}T= \left[ \begin{array}{@{}llll@{}} \displaystyle {T}_{11}&\displaystyle {T}_{12}&\displaystyle \cdots &\displaystyle {T}_{1n}\\ \displaystyle {T}_{21}&\displaystyle {T}_{22}&\displaystyle \cdots &\displaystyle {T}_{2n}\\ \displaystyle \vdots &\displaystyle \vdots &\displaystyle \ddots &\displaystyle \vdots \\ \displaystyle {T}_{n1}&\displaystyle {T}_{n2}&\displaystyle \cdots &\displaystyle {T}_{nn} \end{array} \right] \end{eqnarray*}

Core element:*T*_*ij*_ represents the area/quantity of the *i*th land use type at *t*_1_ transferred to the *j*th land use type at *t*_2_;

Diagonal element*T*_*ii*_: The area/quantity of land use type that remains unchanged from *t*_1_ to *t*_2_;

Off-diagonal element*T*_*ij*_:The area/quantity of land use type that undergoes conversion from *t*_1_ to *t*_2_.

## Results

### Spatial and temporal dynamics of NDVI on the Tibetan Plateau

#### Evaluation of model accuracy

**Figure 4:**
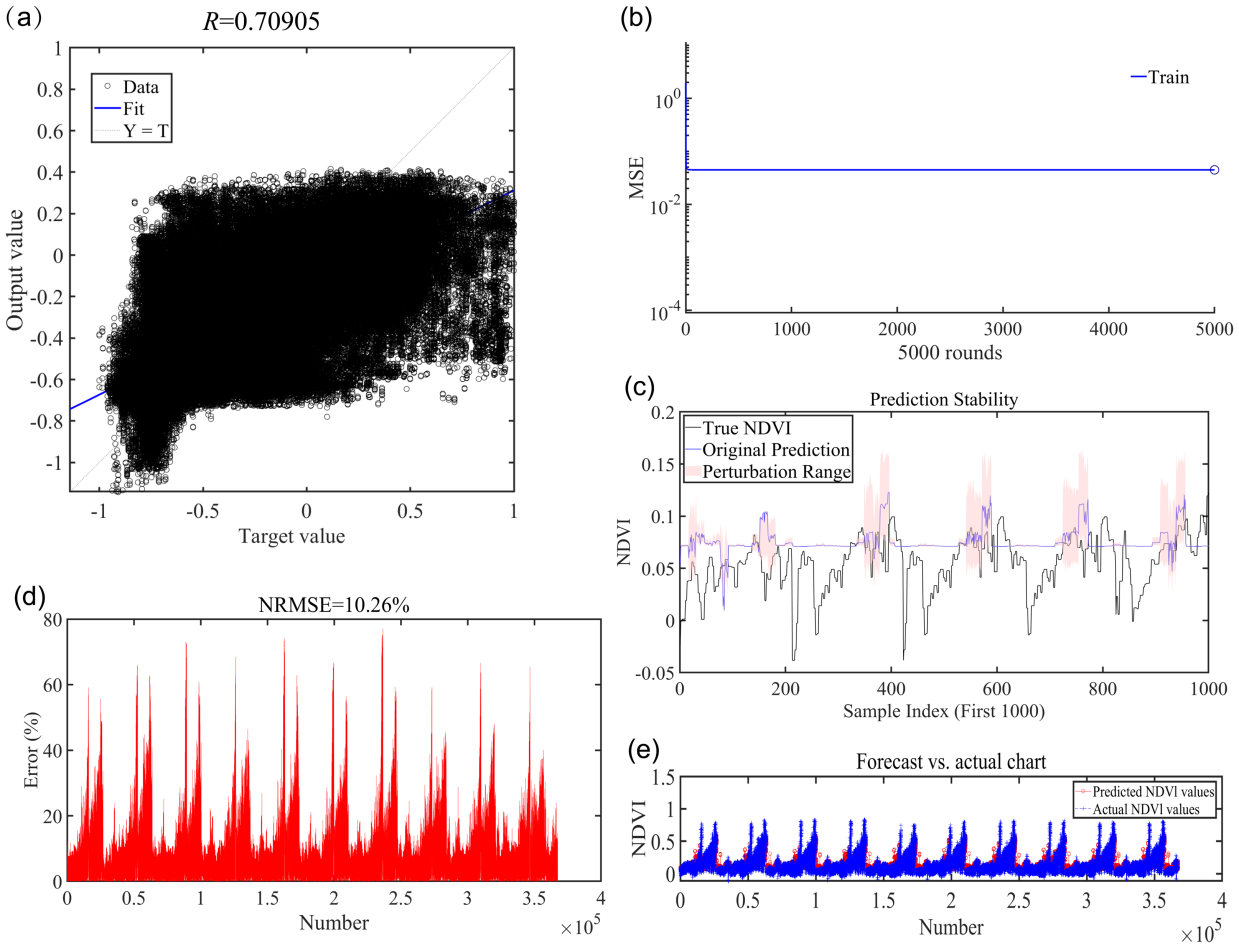
Evaluation of model accuracy. (A) R-value (math). (B) Mean squared error (MSE). (C) Monte Carlo simulations. (D) Normalized root mean squared error (NRMSE). (E) Chart of projected *versus* actual values.

In this study, monthly regression analysis was conducted on the NDVI of the Tibetan Plateau from 2001 to 2010. A total of 367,511 monthly data points were utilized over the 10-year period. The analysis yielded an average R-value of 0.7 across the 12 months, an NRMSE of 10.26%, and an MSE of 0.04. The range of the R value was [−1, 1]. An R-value closer to 1 indicates a strong positive correlation, while one nearing −1 signifies a strong negative correlation. The MSE and NRMSE range was [0, +∞); values approaching 0 indicate greater consistency between the model’s predicted and true values. Therefore, the model exhibited good accuracy. Concurrently, Monte Carlo simulations were performed, yielding the following values: Original MSE: 0.0089; Mean MSE after perturbation: 0.0128; Std of MSE: 0.0000. In the context of Monte Carlo simulation perturbation, model error increased; however, the fluctuation remained minimal, thereby indicating good stability (Fig. 4).

#### Analysis of NDVI spatial and temporal dynamics

**Figure 5:**
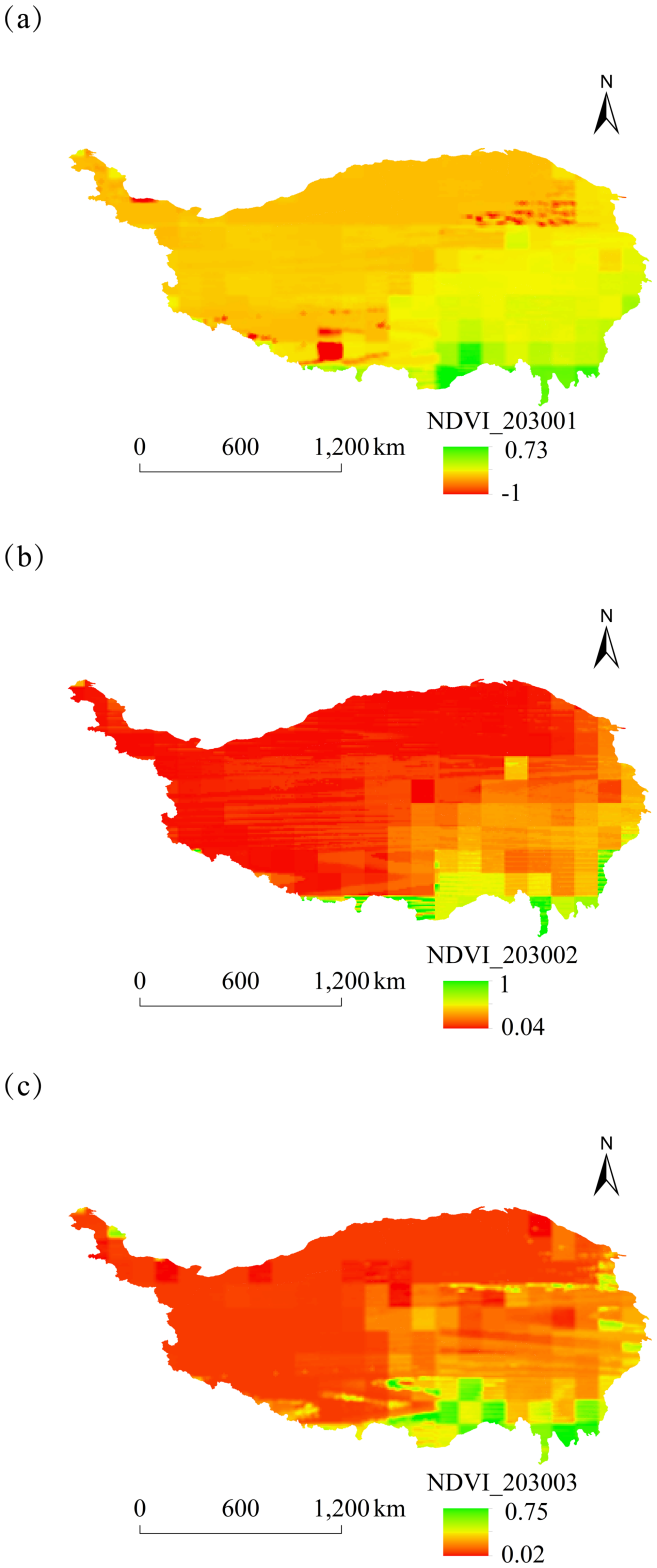
Monthly spatial variation of NDVI on the Tibetan Plateau, China, in 2030. (A) January. (B) February. (C) March.

**Figure 6:**
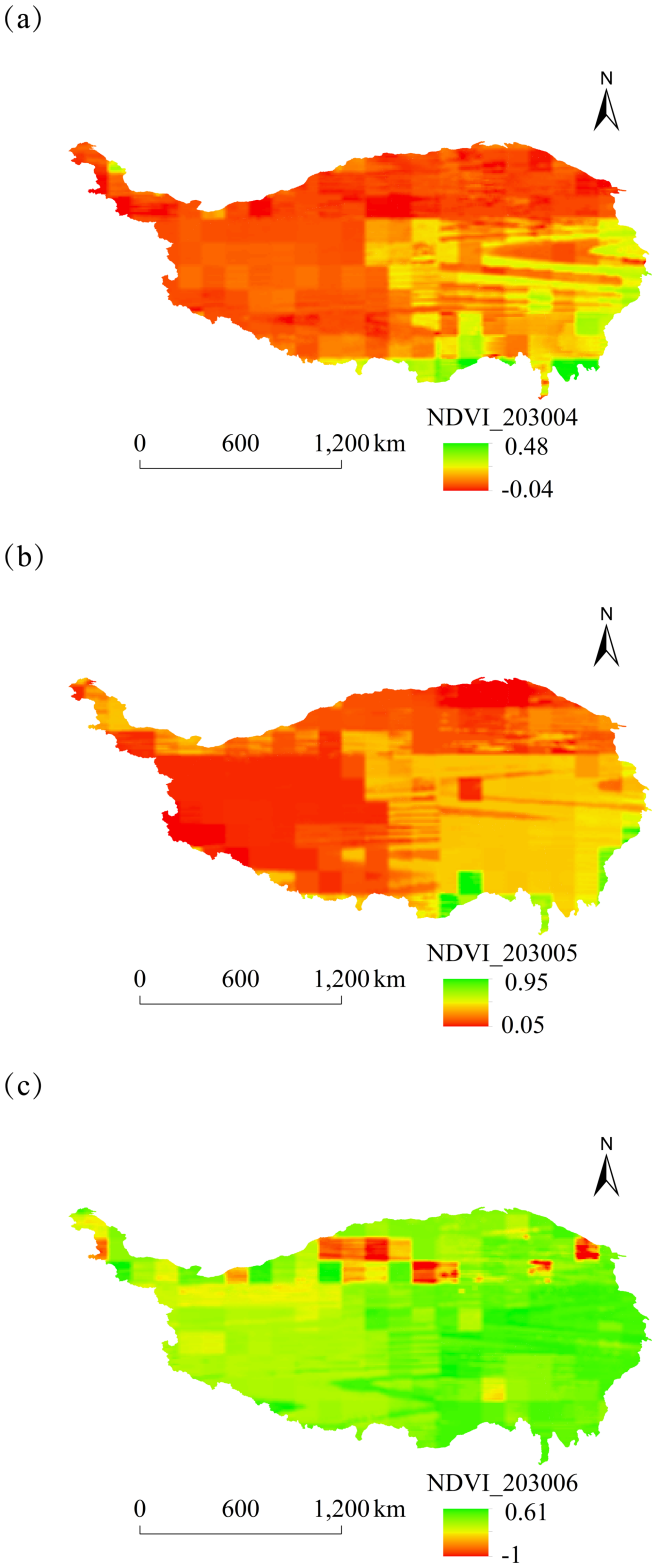
Monthly spatial variation of NDVI on the Tibetan Plateau, China, in 2030. (A) April. (B) May. (C) June.

**Figure 7:**
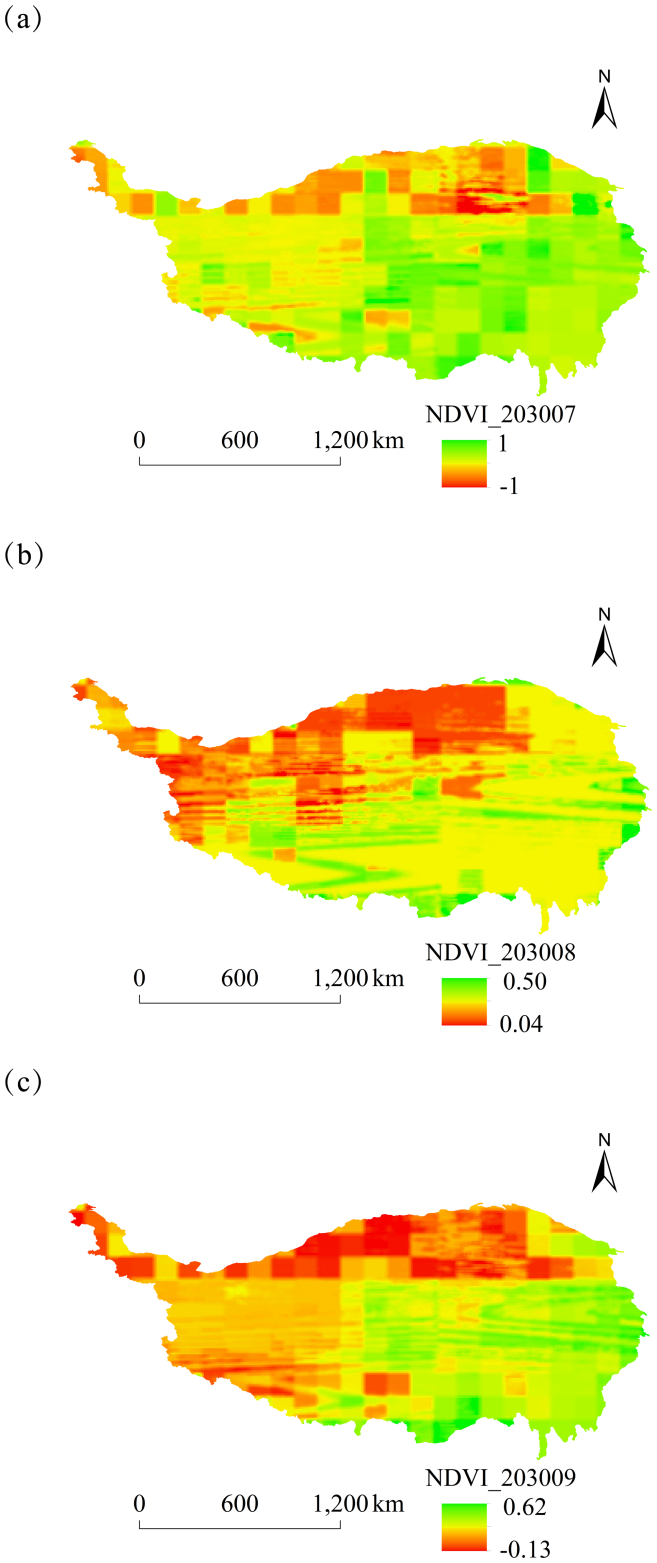
Monthly spatial variation of NDVI on the Tibetan Plateau, China, in 2030. (A) July. (B) August. (C) September.

**Figure 8:**
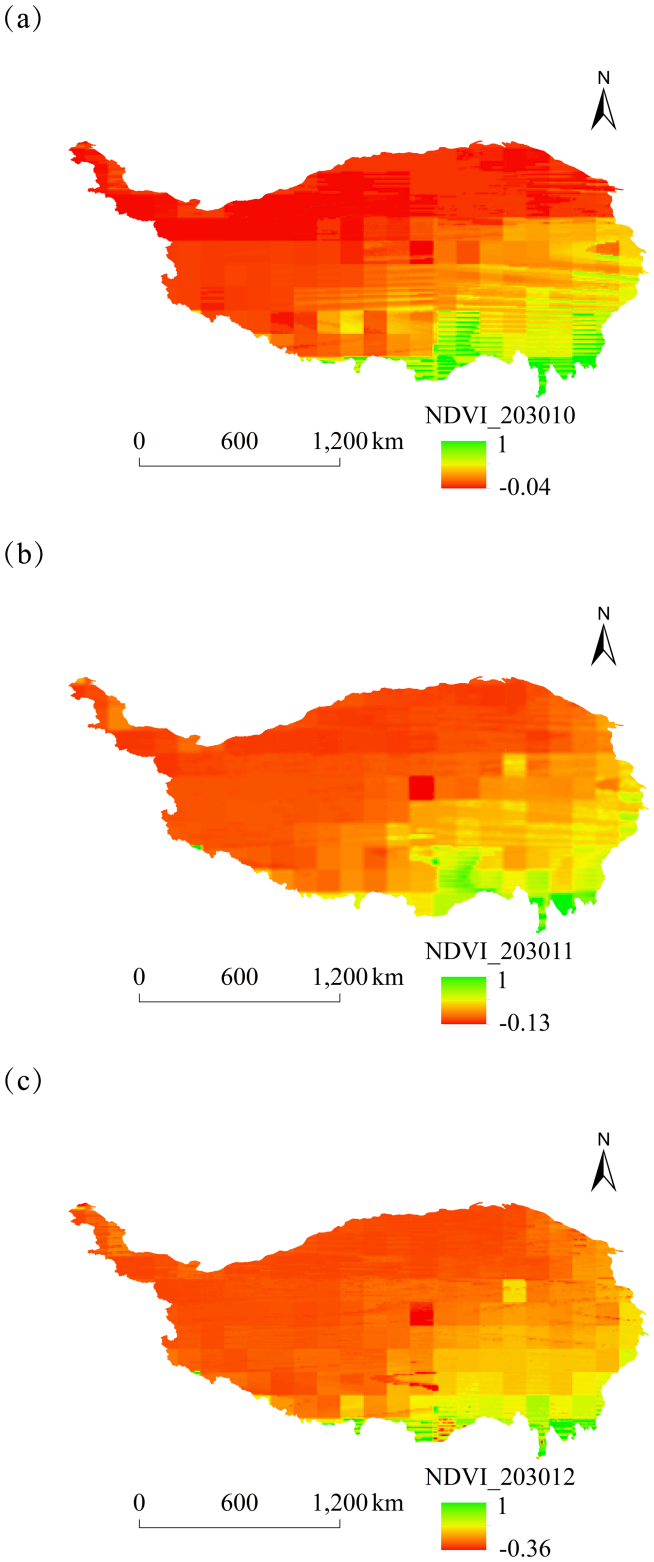
Monthly spatial variation of NDVI on the Tibetan Plateau, China, in 2030. (A) October. (B) November. (C) December.

The spatial distribution of monthly NDVI on the Tibetan Plateau of China from 2025 to 2030 was characterized by high values in the southeast and low values in the northwest. Notable differences in NDVI between these regions were particularly significant in January, May, and September (Figs. 5–8).

The overall spatial distribution of NDVI on the Tibetan Plateau over the next six years showed relatively high NDVI values in June–August and relatively low values in February, October, and November.

**Figure 9:**
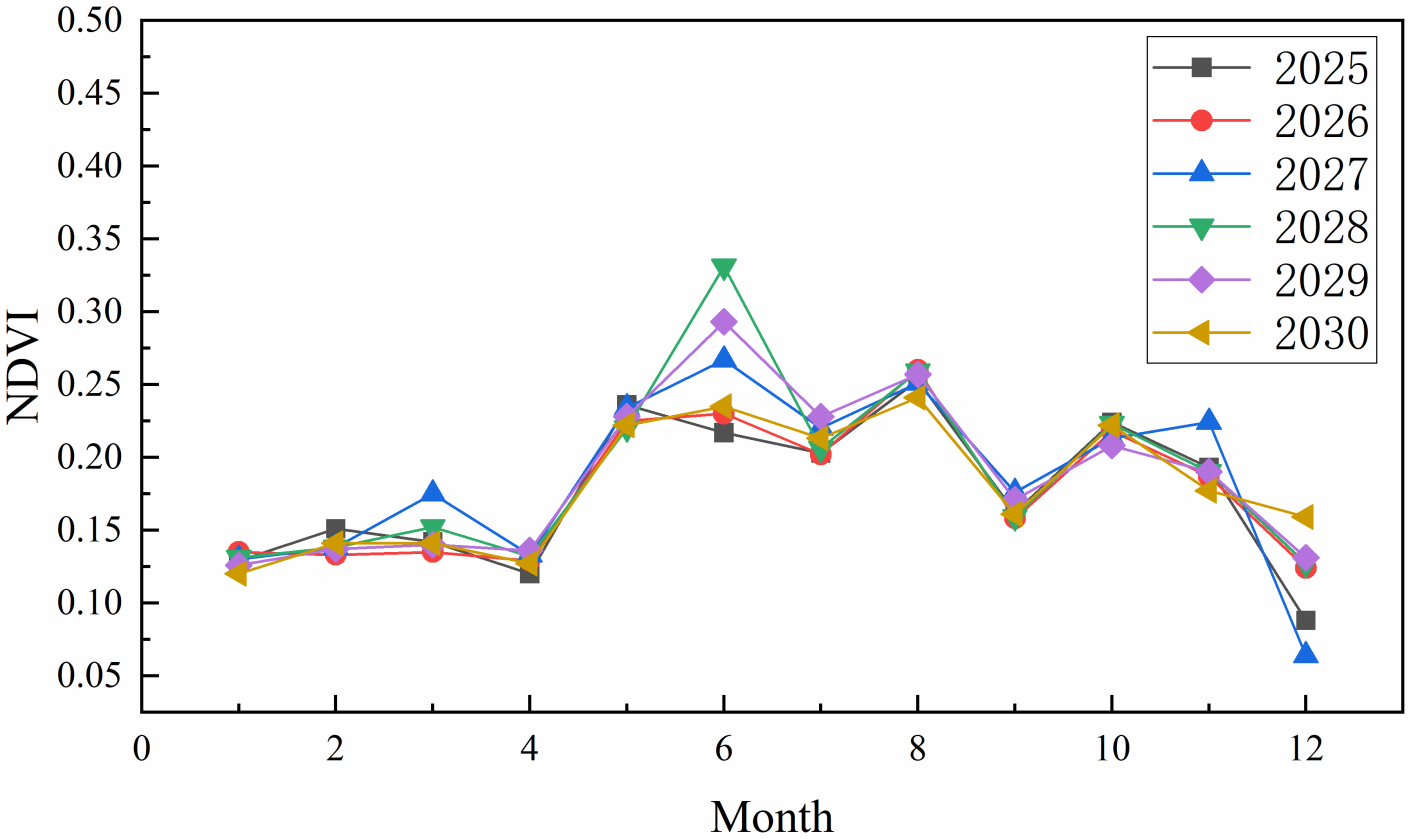
Changes in monthly mean NDVI on the Tibetan Plateau, China, from 2025 to 2030.

The folded plot of monthly mean NDVI changes on the Tibetan Plateau from 2025 to 2030 indicated that the highest monthly mean NDVI values occurred in August for 2025, 2026, and 2030 and June for 2027–2029. The monthly average NDVI minimums for 2025–2028 occurred in December, while those for 2029 and 2030 were recorded in January (Fig. 9). The highest monthly average NDVI value over the six years was 0.331 in June 2028, while the lowest was 0.064 in December 2027.

### Spatial and temporal dynamics of NPP and wetland vegetation carbon sequestration potential on the Tibetan Plateau

**Figure 10:**
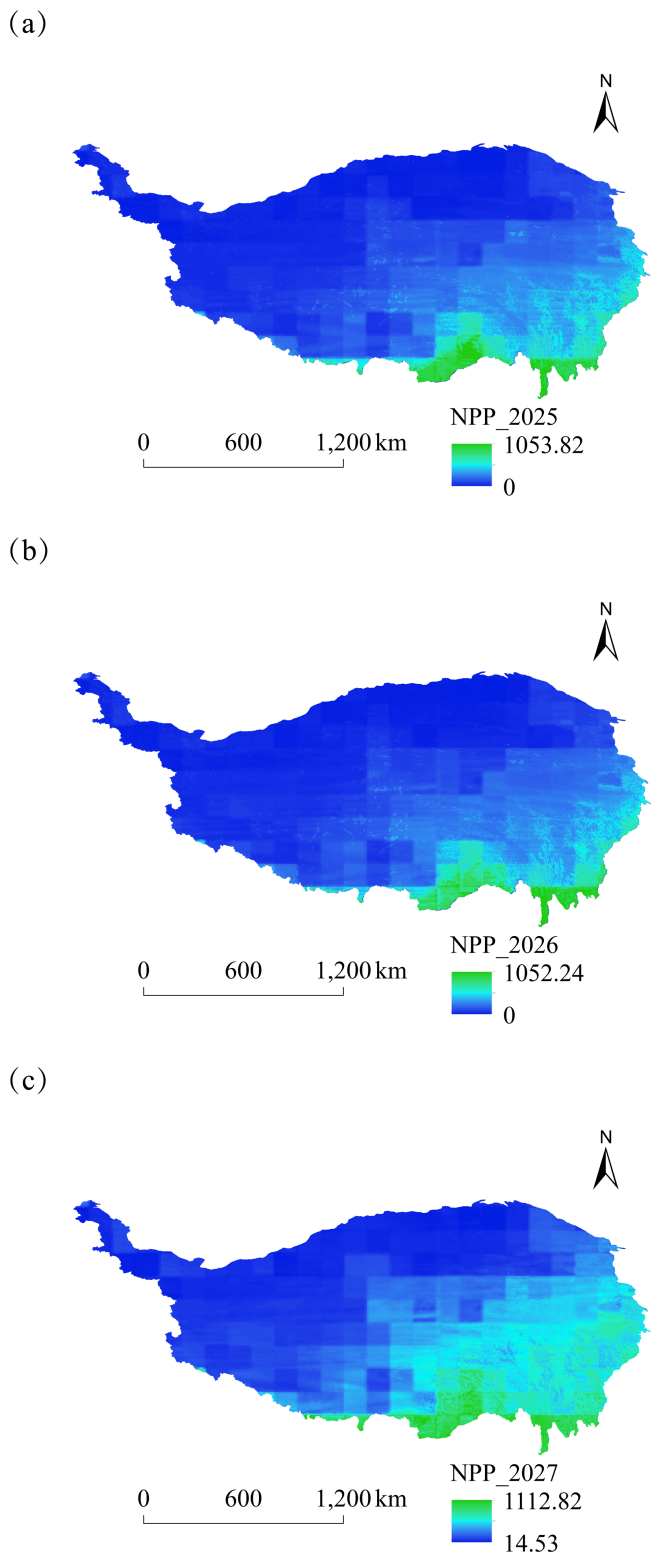
Spatial variation of NPP on the Tibetan Plateau, China, from 2025 to 2027. (A) Spatial and temporal changes in NPP in 2025. (B) Spatial and temporal changes in NPP in 2026. (C) Spatial and temporal changes in NPP in 2027.

**Figure 11:**
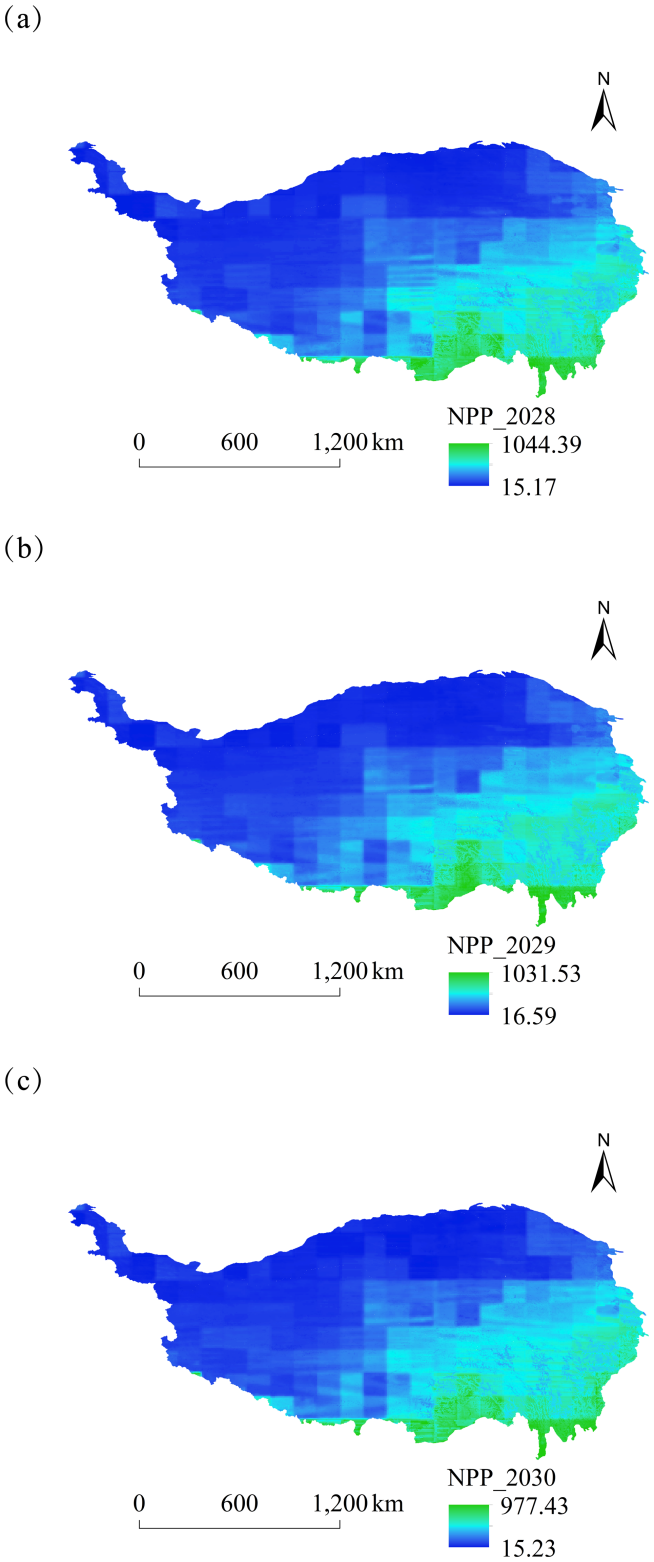
Spatial variation of NPP on the Tibetan Plateau, China, from 2028 to 2030. (A) Spatial and temporal changes in NPP in 2028. (B) Spatial and temporal changes in NPP in 2029. (C) Spatial and temporal changes in NPP in 2030.

In this study, we simulated the spatial and temporal dynamics of NPP on the Tibetan Plateau from 2025 to 2030 based on the CASA model (Figs. 10 and 11). The NPP distribution was generally higher in the southeast than that in the northwest, demonstrating a strong correlation with vegetation cover, topography, and climatic factors. Higher NPP values were observed in Sichuan, Yunnan, and southeastern Xizang on the Tibetan Plateau. The distribution map indicated that the NPP values in Sichuan Province, Yunnan Province, and Southeast Xizang on the Tibetan Plateau increased significantly each year. The dominant land cover types in the Yunnan section, Sichuan section, and southeastern Xizang section of the Qinghai-Tibet Plateau are forestland and grassland (data from 2025): the Yunnan section has 74.1% forestland and 18.7% grassland; the Sichuan section has 37.7% forestland and 55.9% grassland; the southeastern Xizang section has 29.7% forestland and 58.1% grassland. The distribution characteristics of these land cover types are closely related to the spatial heterogeneity of regional net primary productivity (NPP). The average NPP value for 2025 was predicted to be 166.70 gC ⋅ m^−2^ ⋅ a^−1^, while that for 2030 was projected to be 194.73 gC ⋅ m^−2^ ⋅ a^−1^. Therefore, an increasing trend in NPP was observed on the Tibetan Plateau.

**Figure 12:**
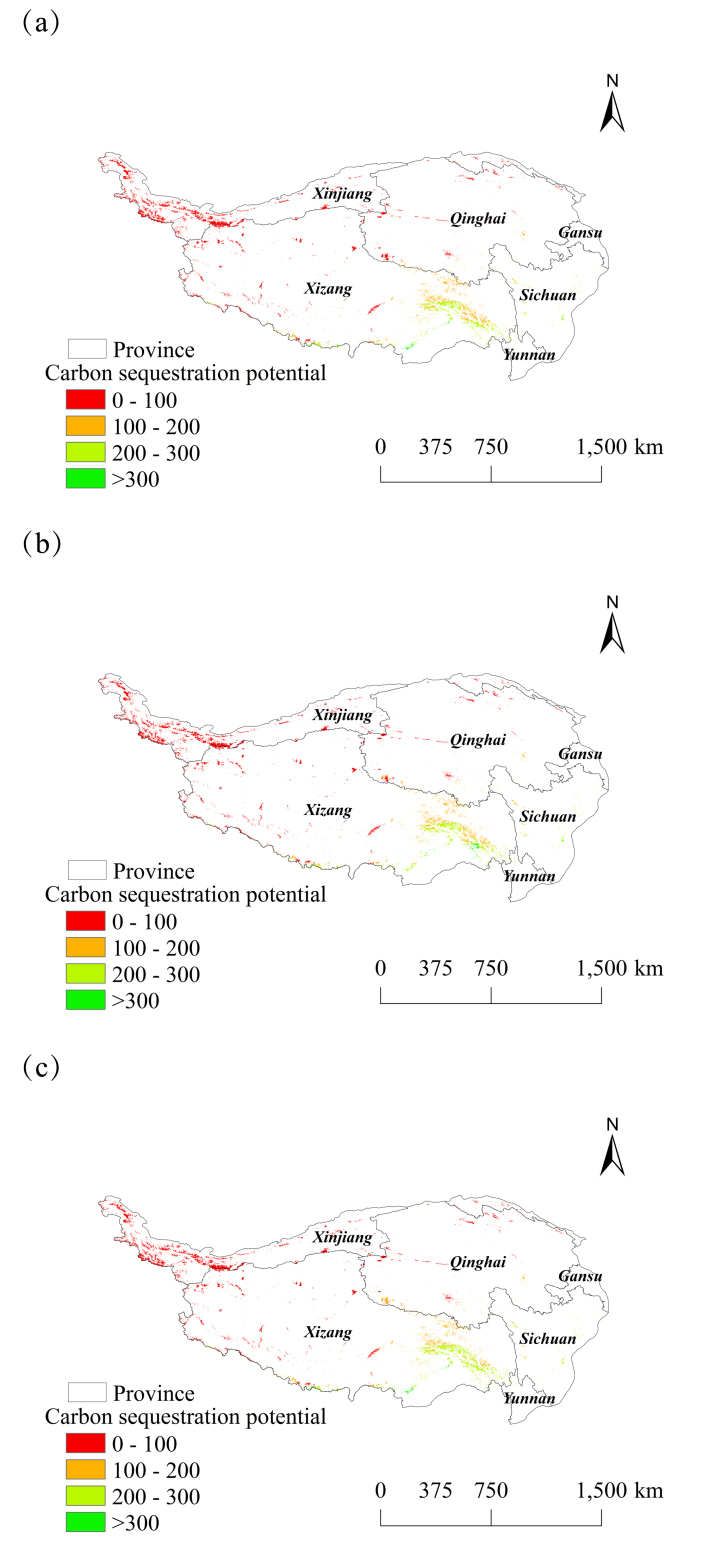
Spatial changes in carbon sequestration potential of wetlands on the Tibetan Plateau from 2025 to 2027. (A) 2025. (B) 2026. (C) 2027.

**Figure 13:**
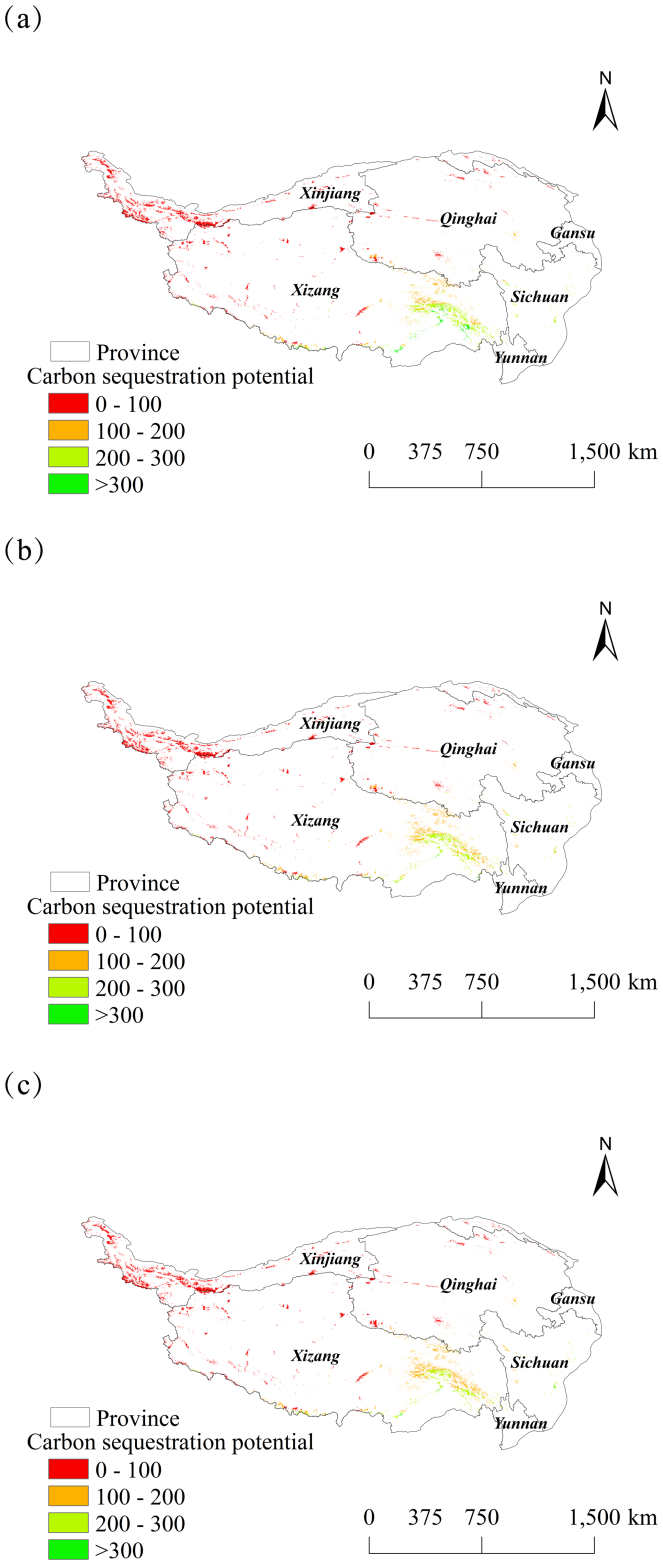
Spatial changes in carbon sequestration potential of wetlands on the Tibetan Plateau from 2028 to 2030. (A) 2028. (B) 2029. (C) 2030.

In this study, wetlands on the Tibetan Plateau were extracted, the carbon sequestration capacity of wetlands was predicted from 2025 to 2030, and a spatial distribution map was created (Figs. 12 and 13). The carbon sequestration potential of these wetlands was primarily concentrated in the range of 0–100 gC ⋅ m^−2^ ⋅ a^−1^. Wetlands with a high carbon sink potential were concentrated near the Palung Tsangpo, the largest tributary of the Yarlung Tsangpo River. A slight increase in the carbon sequestration potential over time is illustrated in the distribution plot. The lowest value in six years was 7.00 gC ⋅ m^−2^ ⋅ a^−1^ in 2028, while the highest value was 472.71 gC ⋅ m^−2^ ⋅ a^−1^ in 2027.

### Analysis of the impact of land type on NPP

In this study, the land-use shift of the Tibetan Plateau for 2020–2030 was calculated based on the land-use shift matrix (Table 2). According to the shift, the predominant land type in 2020 and 2030 was grassland (1,745,555.87 and 1,727,634.96 km^2^, respectively), constituting 64.39% and 63.73% of the total Tibetan Plateau area, respectively. The smallest land type area was the urban area at 1,408.66 km^2^ in 2030, reflecting an increase of 184.30% compared to that observed in 2020 and accounting for 0.05% of the entire Tibetan Plateau. The forest area increased by 1,270.53 km^2^, reflecting a growth rate of 0.43%. This increase included the conversion of 1,241.25 km^2^ of cultivated land to forest land, representing 0.42% of the total forest land and 1.71% of the total cultivated land. The grassland was 17,920.91 km^2^, reflecting a reduction rate of 1.03%. This reduction primarily resulted from the conversion of grassland to barren land and forests, which accounted for 2.95% and 0.71% of the total area of grassland, respectively. Meanwhile, the conversion of cultivated land to grassland measured 5,194.98 km^2^, representing 0.30% of the total grassland area and 7.14% of the total cultivated land area. The above data indicate that the Tibetan Plateau implemented the initiative of converting farmland back to forests, resulting in the reestablishment of forests, grasslands, and other vegetation on previously cultivated land.

**Table 2:** Land-use transformation on the Tibetan Plateau, China, 2020–2030 (km^2^).

Year	Land use type	**2030**						
		**Grassland**	**Urban**	**Cropland**	**Forest**	**Water**	**Barren**	**Sum**
2020	Grassland	1,674,080.00	621.07	4,410.37	12,466.30	2,512.63	51,465.50	1,745,555.87
	Urban	20.20	342.98	19.65	18.95	0.00	93.71	495.48
	Cropland	5,194.98	231.40	65,535.90	1,241.25	11.18	568.94	72,783.66
	Forest	12,062.90	134.19	1,780.98	278,227.00	69.71	468.88	292,743.65
	Water	394.48	1.24	58.10	285.74	44,509.60	1,652.62	46,901.79
	Barren	35,882.40	77.79	290.11	1,774.93	1,429.83	513,061.00	552,516.05
	Sum	1,727,634.96	1,408.66	72,095.10	294,014.18	48,532.95	567,310.65	2,710,996.50

The average annual NPP values for the Tibetan Plateau from 2025 to 2030 were analyzed based on different land types. The highest value recorded was 424.58 gC ⋅ m^−2^ ⋅ a^−1^ for forests, while the lowest observed was 75.83 gC ⋅ m^−2^ ⋅ a^−1^ for unutilized land. Meanwhile, the rates of change in the six-year period were the highest for forests and lowest for water bodies at 22.02 and 0.19 gC ⋅ m^−2^ ⋅ a^−1^, respectively.

### Analysis of the impact of climatic factors on NPP

**Figure 14:**
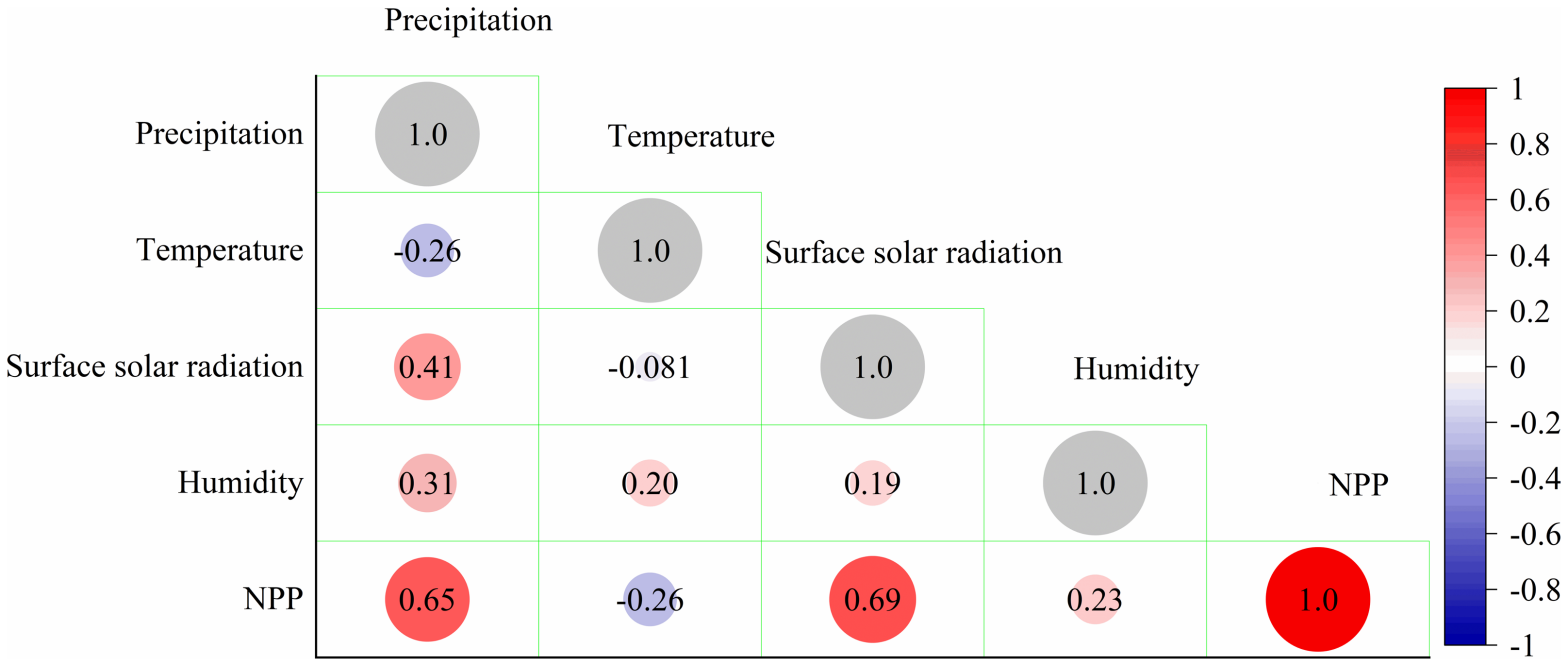
Correlation between climatic factors and NPP.

An in-depth analysis of the effects of climatic factors on NPP is essential for understanding the response mechanisms of carbon sequestration in response to global changes and for predicting the evolutionary trends of carbon sequestration potential. Spearman’s correlation analyses were conducted for four meteorological factors: precipitation, temperature, surface solar radiation, and relative humidity. Our findings show significant positive correlations between precipitation and both surface solar radiation and NPP, with correlation coefficients of 0.65 and 0.69, respectively (Fig. 14). Temperature exhibited a weak negative correlation of −0.26, indicating a complex nonlinear relationship from the perspective of temperature. Temperatures that fall outside the appropriate range for the plant can adversely affect its cellular structure and decrease NPP. Air humidity was weakly positively correlated at 0.23 and indirectly influenced NPP primarily through its effect on plant transpiration. Maintaining appropriate humidity is essential for plant water balance, minimizing water loss, and promoting plant growth. Low humidity can induce plant water deficits and inhibit photosynthesis, whereas excessive humidity may increase the risk of pests and diseases, thereby compromising plant health and subsequently affecting NPP.

## Discussion

### Characteristics of spatial and temporal variations in NDVI

From the time scale, the overall NDVI of the Tibetan Plateau exhibited a fluctuating upward trend over the subsequent six years. This trend reflects a gradual improvement in vegetation cover, aligning with findings that the aboveground biomass of grassland increased during the study period. Moreover, a future increasing trend in NDVI can be inferred to a certain extent (Liu et al., 2017). The highest NDVI values are concentrated from June to August as the warm and humid climate extends the plant growing season. Increased precipitation provides adequate water and creates favorable conditions for vegetation growth. Consequently, the range of vegetation growth is constantly expanding, and the NDVI exhibits a synchronous increase (Wang et al., 2023b). In particular, the high-altitude areas of the central and southwestern Tibetan Plateau improve significantly in terms of vegetation condition (Wei et al., 2022).

In terms of spatial distribution, the NDVI of the Tibetan Plateau exhibits notable regional characteristics. The southeastern part of the plateau, with excellent thermal and hydrological conditions, has emerged as a concentration of forest and scrub vegetation, exhibiting high NDVI values and vigorous vegetation growth. In contrast, the northwestern part of the country is dominated by alpine deserts and grasslands, with an arid and cold local climate, low NDVI values, and sparse vegetation cover. For example, the Hengduan Mountains are greatly influenced by the southwest monsoon, with abundant precipitation, complex mountain formations, intense terrain undulation, and an extremely prominent vertical stratification of vegetation. As the altitude increases, the NDVI shows a consistent gradient owing to variations in vegetation types (Yang et al 2024).

### Characterization of spatial and temporal changes in NPP

The spatial distribution of NPP on the Tibetan Plateau exhibits a typical landscape pattern of “patch-corridor-matrix.” At the macro level, a gradient characterized by high values in the southeast and low values in the northwest is shaped by hydrothermal conditions (Zhang, Hu & Zou, 2021). The micro-scale features a complex “mosaic” shape of NPP due to the interplay of topography, soil types, and vegetation patches. For example, deep-cut river valleys in the alpine canyon area serve as water vapor pathways, exhibiting a marked difference in NPP between the valley floor and adjacent slopes, which display a corridor-like distribution (Guo et al., 2020). The NPP on the plateau surface varies among different vegetation type patches, including alpine meadows and alpine scrubs, each exhibiting distinct characteristics that collectively influence the substrate for the spatial NPP distribution.

Over the next six years, the NPP of the Tibetan Plateau exhibited a fluctuating upward trend; however, significant variations were observed among different vegetation types and ecological function areas, with forest land demonstrating a higher NPP value than that of grassland. Numerous scholars have examined the trend of NPP changes on the Tibetan Plateau throughout history; for example, Yu et al. (2021) analyzed the NPP of grasslands on the Tibetan Plateau during the historical period and demonstrated that the NPP of various vegetation types was influenced by climate and human activities in distinct manners (Yu et al., 2021). In a comprehensive historical context, future changes will occur in closer succession.

### Factors affecting NPP

In this study, the effects of land type and climatic conditions on NPP in the Tibetan Plateau were analyzed separately. Woodlands are primarily found at the edges of plateaus and within certain river valleys. These areas exhibit relatively good hydrothermal conditions and superior soil fertility and water- and fertilizer-holding capacities than those observed for grasslands. Trees exhibit increased height, biomass, photosynthesis, and NPP (Xu et al., 2020). The higher NPP values and rates of change in forests play a key role in the carbon cycle across the entire Tibetan Plateau. As an important carbon sink, forests can absorb large amounts of carbon dioxide and play an important role in mitigating the greenhouse effect. Therefore, protection and rational utilization of forests should be strengthened, while scientific forest management plans should be formulated to reduce the adverse impacts of human activities on their ecosystems. Grassland covers a vast area of the Tibetan Plateau and represents a primary land type. Its soil is relatively poor, characterized by a thick grassroots layer that retains water and nutrients to some extent. However, this land type is limited by low temperatures and a short growing season, resulting in vegetation predominantly composed of herbaceous plants with comparatively low NPP. However, grassland ecosystems exhibit relative stability, importantly contribute to regional ecological balance, and enhance the NPP of the entire ecosystem through gradual material cycling and energy conversion (Xiong et al., 2021).

Precipitation and light greatly affect NPP. Correlation analysis indicated strong positive correlations between precipitation and solar radiation with NPP. Precipitation is a key climatic factor that directly affects water availability for plants. Adequate precipitation provides the water required for plant photosynthesis, maintains cell expansion pressure, and facilitates the normal opening and closing of stomata for gas exchange, thereby enhancing NPP. In arid regions, increased precipitation substantially improves vegetation growth and cover, thereby improving NPP. Solar radiation serves as the energy source for photosynthesis, with its intensity and duration directly influencing the energy available to plants. Stronger solar radiation enhances energy availability for photosynthesis, resulting in greater synthesis of photosynthetic products and an elevation in NPP. Temperature greatly influences NPP. However, NPP does not increase or decrease with increasing temperature; instead, it tends to increase within suitable temperature ranges. This demonstrates that the temperature falls within a specific threshold range conducive to NPP (Ma et al., 2021).

### Carbon sequestration potential of wetlands

The unique alpine climatic conditions of wetlands on the Tibetan Plateau result in slow decomposition of vegetative debris, facilitating the accumulation of substantial organic matter and formation of a deep peat layer. These peat layers are important carriers of carbon in wetlands and store large amounts of SOC. This study concluded that wetlands with high carbon sink potential on the Tibetan Plateau from 2025 to 2030 are primarily concentrated near the Palung Tsangpo, the largest tributary of the Yarlung Tsangpo River. The wetland carbon sinks also increased during this period. A previous study on carbon sequestration in alpine grasslands on the Tibetan Plateau during historical times has also been conducted (Wang et al., 2023b). This study, while not specifically focused on wetlands of the Tibetan Plateau, suggests that these wetlands, as components of alpine grasslands, are expected to exhibit an upward trend in their carbon sequestration potential in the future, thereby aligning with the overall increase in carbon sequestration within alpine grasslands.

### Limitations and uncertainty analysis

The CASA model, although widely used in NPP estimation, encountered challenges related to fine-scale application and parameterization (Zhang et al., 2025b). The method employed in this study for predicting wetland carbon sequestration potential from 2025 to 2030 had certain limitations in its application scope: it is restricted to assessing carbon sequestration potential in wetland ecosystems. Therefore, the findings of this research were confined to wetland ecosystems themselves.

In the NDVI estimation of this study, kriging interpolation was employed to address the spatial discontinuity of discrete data output by the BP neural network (to meet the calculation requirements at the wetland patch scale, with the process strictly adhering to geostatistical principles). However, the combination of these two methods involves error propagation effects: the inherent biases in the input data of the BP neural network are transmitted through interlayer weight propagation, and then superimposed with the errors generated by kriging interpolation due to vegetation spatial heterogeneity and uneven sampling density, exerting a certain impact on the NDVI estimation accuracy. Additionally, the future climate change data used for predicting NDVI changes in this study is based on model projections. Constrained by factors such as model structure and parameterization schemes, these data inherently possess uncertainties, which may affect the precision of predicting future NDVI changes, constituting another limitation of this study.

Future research should focus on integrating multi-source remote sensing data and optimizing existing prediction methods to improve accuracy. Additionally, the carbon sequestration assessment framework should be expanded to other key ecosystems on the Qinghai-Tibet Plateau (*e.g.*, grasslands, forests, and alpine meadows) to develop a comprehensive regional carbon sequestration model.

## Conclusions

Given the high altitude and large temperature fluctuations that characterize the Qinghai-Tibet Plateau, this study combined the nonlinear fitting advantages of BP neural networks with Kriging interpolation for the first time to construct an NDVI prediction framework that was suitable for this region. The spatio-temporal variation characteristics of NDVI on the Qinghai-Tibet Plateau from 2025 to 2030 were predicted. The CASA model utilized NDVI as the input layer to estimate the spatio-temporal variation characteristics of NPP and wetlands on the Qinghai-Tibet Plateau from 2025 to 2030. This study aimed to investigate the effects of various land-use types and climatic factors on NPP values. The following conclusions were drawn:

(1) The monthly NDVI distribution on the Tibetan Plateau was generally higher in the southeast than that in the northwest. Temperature affected the NDVI values.

(2) The NPP spatial distribution on the Tibetan Plateau exhibited a characteristic “patch-corridor-matrix” landscape pattern, with a maximum value of 1,112.82 gC ⋅ m^−2^ ⋅ a^−1^ observed over six years. The rate of increase demonstrated fluctuations.

(3) The carbon sequestration potential of wetlands on the Tibetan Plateau ranged from 0 to 100 gC ⋅ m^−2^ ⋅ a^−1^. Wetlands with high carbon sequestration potential were predominantly concentrated near the Palong Tsangpo, the largest tributary of the Yarlung Tsangpo River.

(4) Vegetation NPP varied significantly among different land-use types on the Tibetan Plateau, with forested land demonstrating the highest mean NPP value and the most substantial rate of change.

(5) Precipitation and solar radiation were significantly and positively correlated with NPP; temperature was weakly and negatively correlated, while humidity was weakly and positively correlated. A complex nonlinear relationship was observed between meteorological conditions and NPP, with a threshold value determining the influence of these conditions on NPP.

These findings offer critical support for ecological conservation and climate mitigation on the Tibetan Plateau:

On the one hand, identifying regions with strong carbon sequestration potential (*e.g.*, vegetation areas exhibiting increasing trends in NDVI and NPP) allows for the formulation of targeted strategies for prioritizing ecological conservation. This includes designating key protected areas and optimizing ecological restoration plans, ultimately enhancing the carbon sequestration function of ecosystems. On the other hand, considering the potential impacts of land-use changes on carbon sequestration (*e.g.*, risks of potential carbon loss due to construction land expansion or vegetation degradation), proactive climate mitigation measures can be preemptively developed (*e.g.*, regulating the intensity of land development and promoting carbon-neutral land management models), thereby providing scientific support for formulating regional policies aimed at addressing climate change.

## Supplemental Information

10.7717/peerj.20758/supp-1 Supplemental Information 1 Supplementary figures

## Additional Information and Declarations

### Data Availability

The following information was supplied regarding data availability: The data is available at FigShare: CAO, Liang (2025). Raw Data. figshare. Dataset. https://doi.org/10.6084/m9.figshare.28877438.v2.
