# Supplementary material for: Temporal and spatial dynamics of Net Primary Productivity and prediction of wetland carbon sequestration potential on the Tibetan Plateau
Source: PeerJ. 2026 Feb 4;14:e20758. doi: 10.7717/peerj.20758 (PMC12882731; doi:10.7717/peerj.20758)
Supplement: Supplemental Information 1 [file peerj-14-20758-s001.docx]

Supplementary Material

# Supplementary Figures

To predict NDVI and NPP from 2025 to 2030, a combined model framework was developed. First, Kriging interpolation processed historical discrete NDVI data, filling spatial gaps to generate a continuous dataset. Second, a 3-layer BP neural network was constructed: the input layer used normalized data of HUR (relative humidity), MRSOL (soil moisture), PRE (precipitation), TEM (temperature), and TSL (soil temperature); the hidden layers had 5 and 7 neurons, respectively; and the output layer was NDVI. The network was trained via backpropagation (learning rate 0.05, max 5000 iterations, target error 0.00065) on full historical data, then used to predict 2025–2030 NDVI from new input features. Finally, predicted NDVI was input into the CASA model, combined with environmental parameters, to predict NPP for the same period.

This framework forms a complete prediction chain from basic data to target parameters, integrating data repair, multi-feature modeling, and ecological estimation

**Fig. 1.** BP neural network structure diagram.
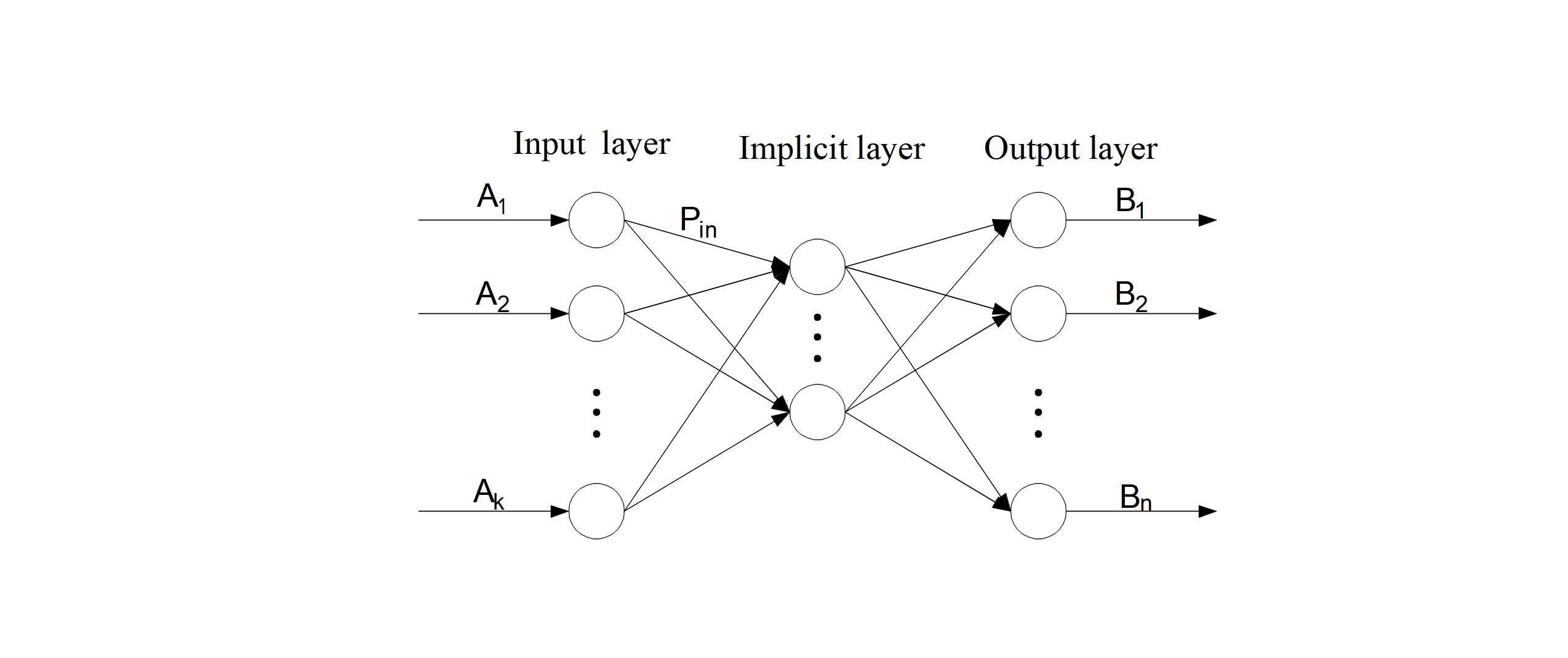


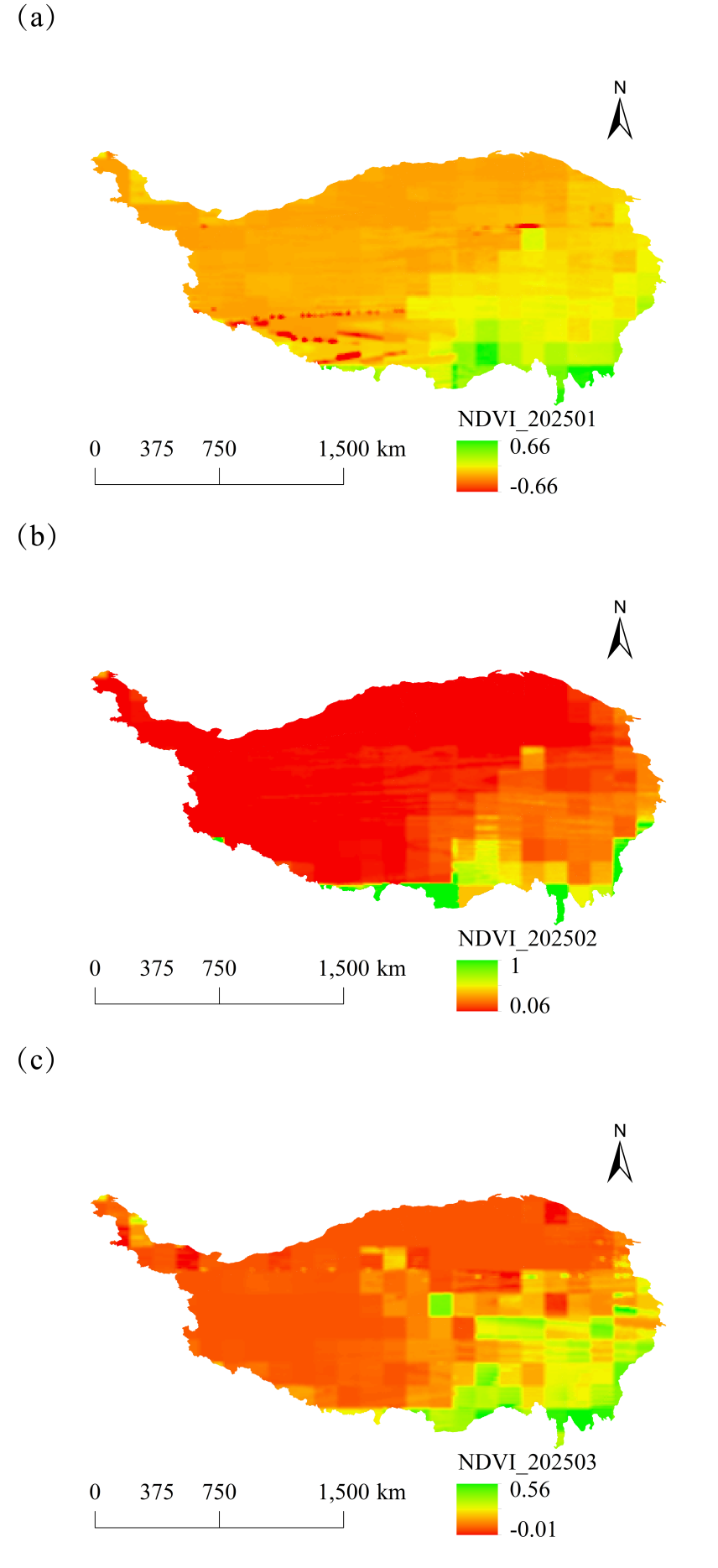
**Fig. 2.** Monthly spatial variation of NDVI on the Tibetan Plateau, China, in 2025. (a): January, (b): February, (c): March.

**
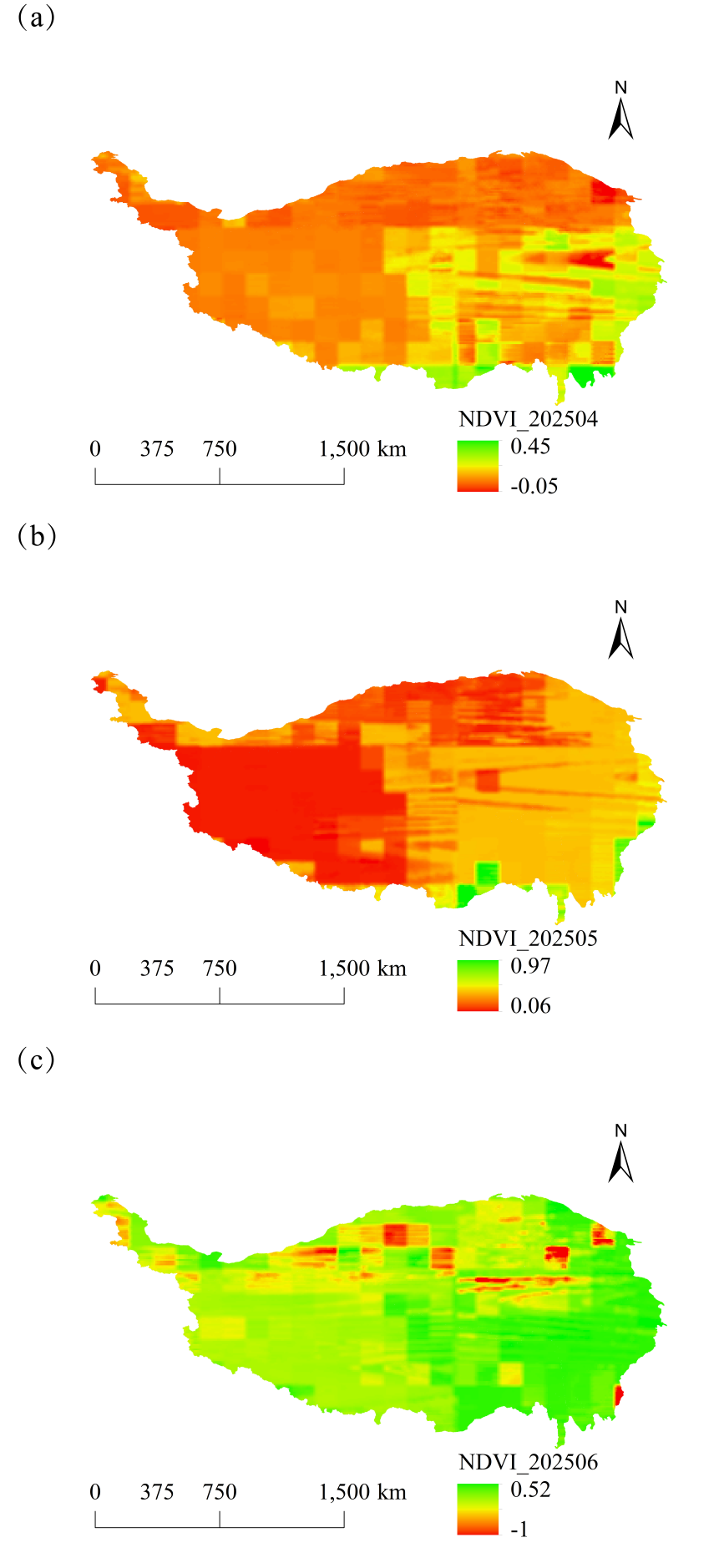
**

**Fig. 3.** Monthly spatial variation of NDVI on the Tibetan Plateau, China, in 2025. (a): April, (b): May, (c): June.


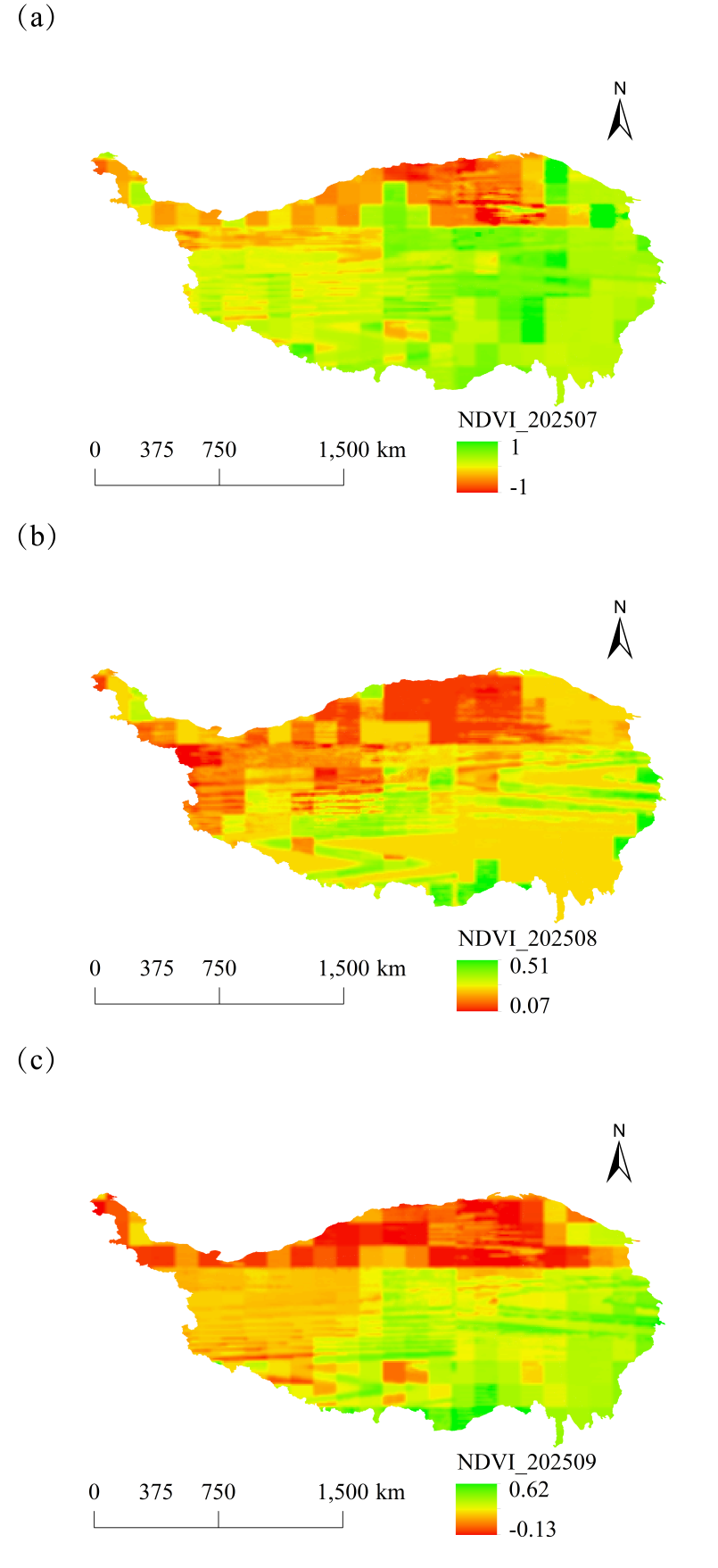


**Fig. 4.** Monthly spatial variation of NDVI on the Tibetan Plateau, China, in 2025. (a): July, (b): August, (c): September.


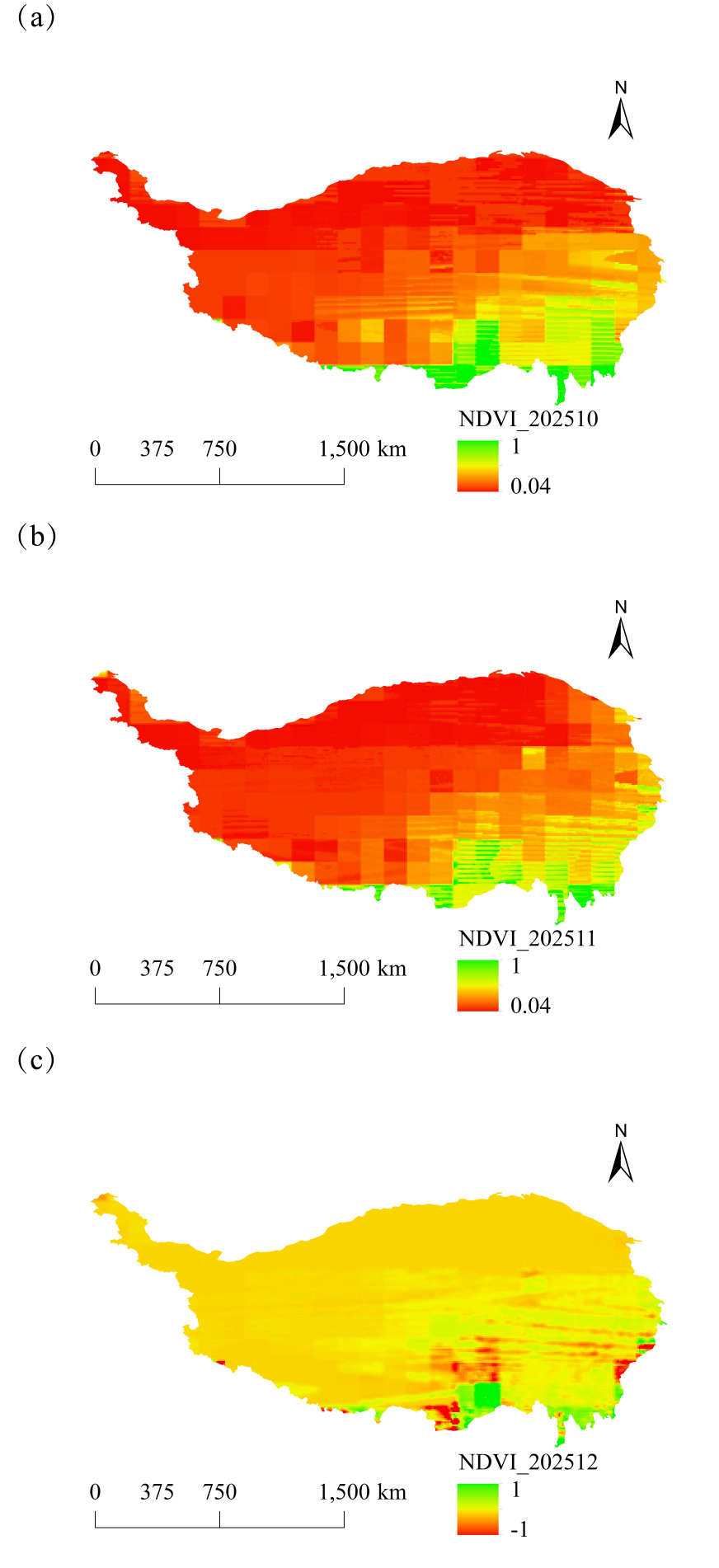
**Fig. 5.** Monthly spatial variation of NDVI on the Tibetan Plateau, China, in 2025. (a): October, (b): November, (c): December.

**
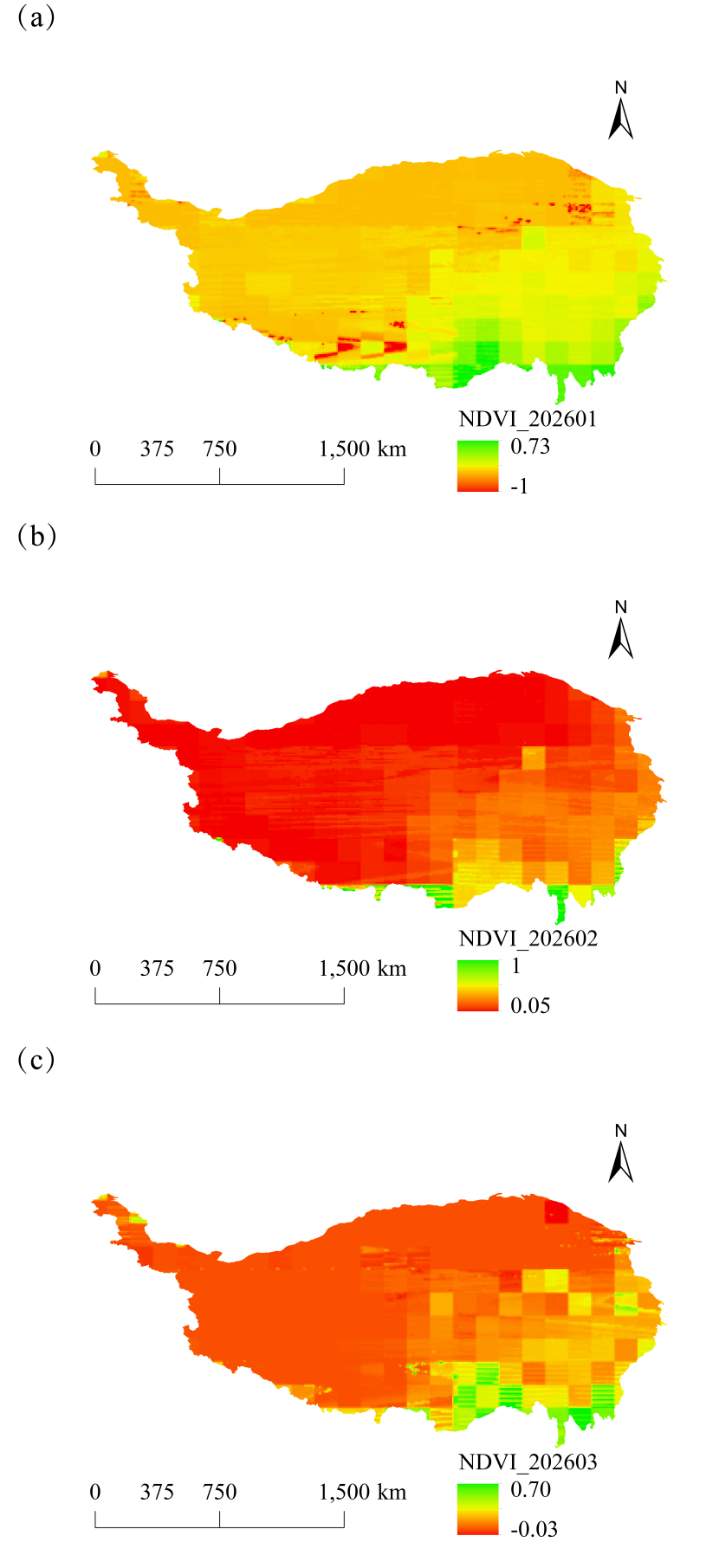
**

**Fig. 6.** Monthly spatial variation of NDVI on the Tibetan Plateau, China, in 2026. (a): January, (b): February, (c): March.

**
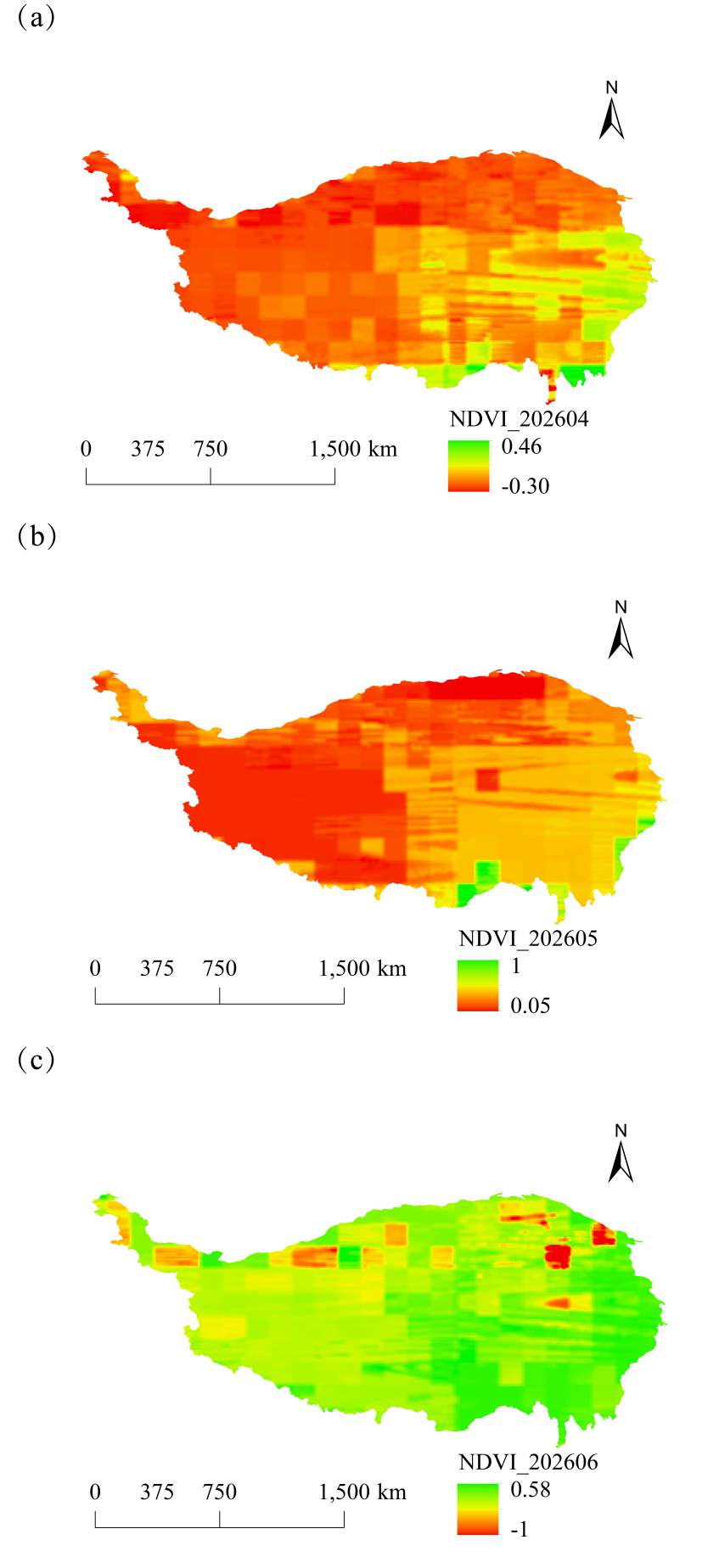
**

**Fig. 7.** Monthly spatial variation of NDVI on the Tibetan Plateau, China, in 2026. (a): April, (b): May, (c): June.

**
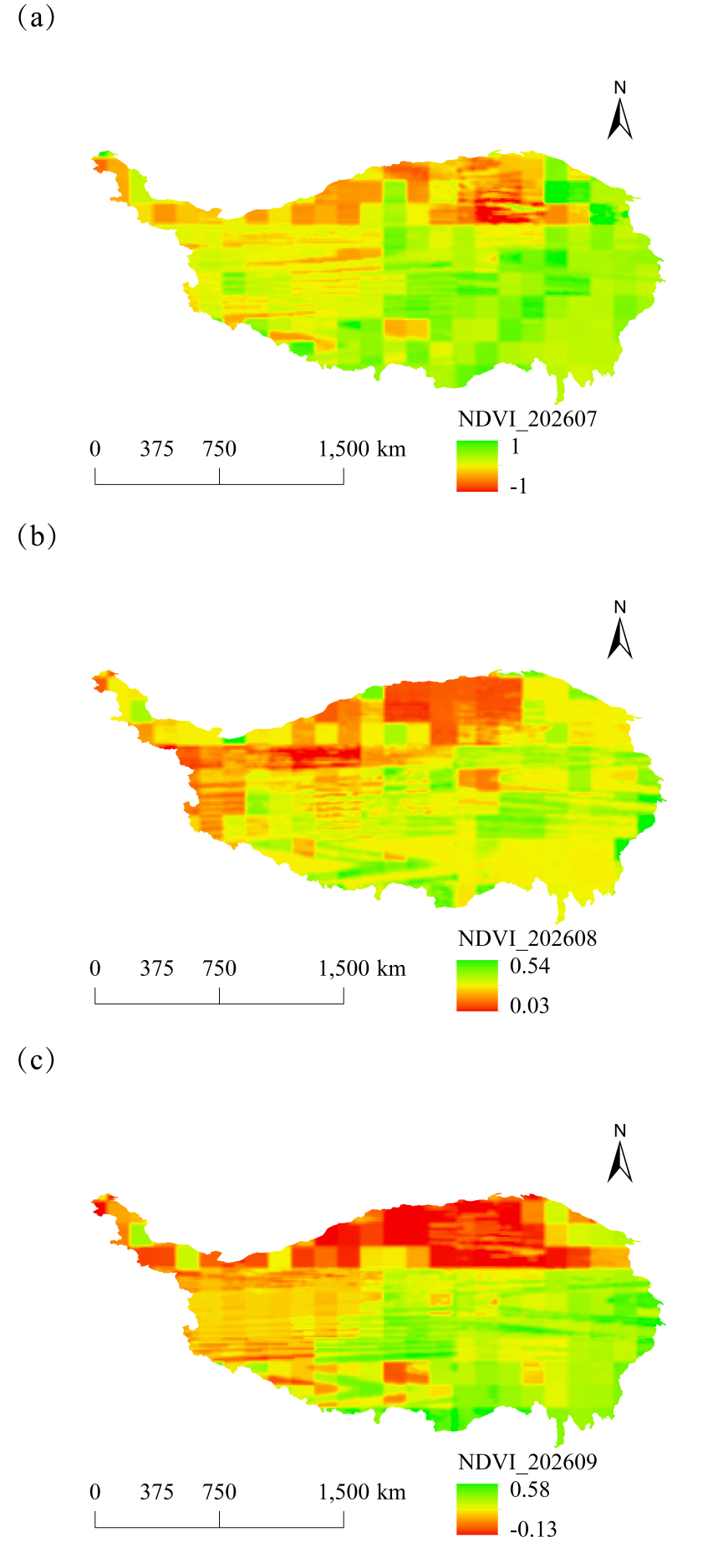
Fig. 8.** Monthly spatial variation of NDVI on the Tibetan Plateau, China, in 2026. (a): July, (b): August, (c): September.

**
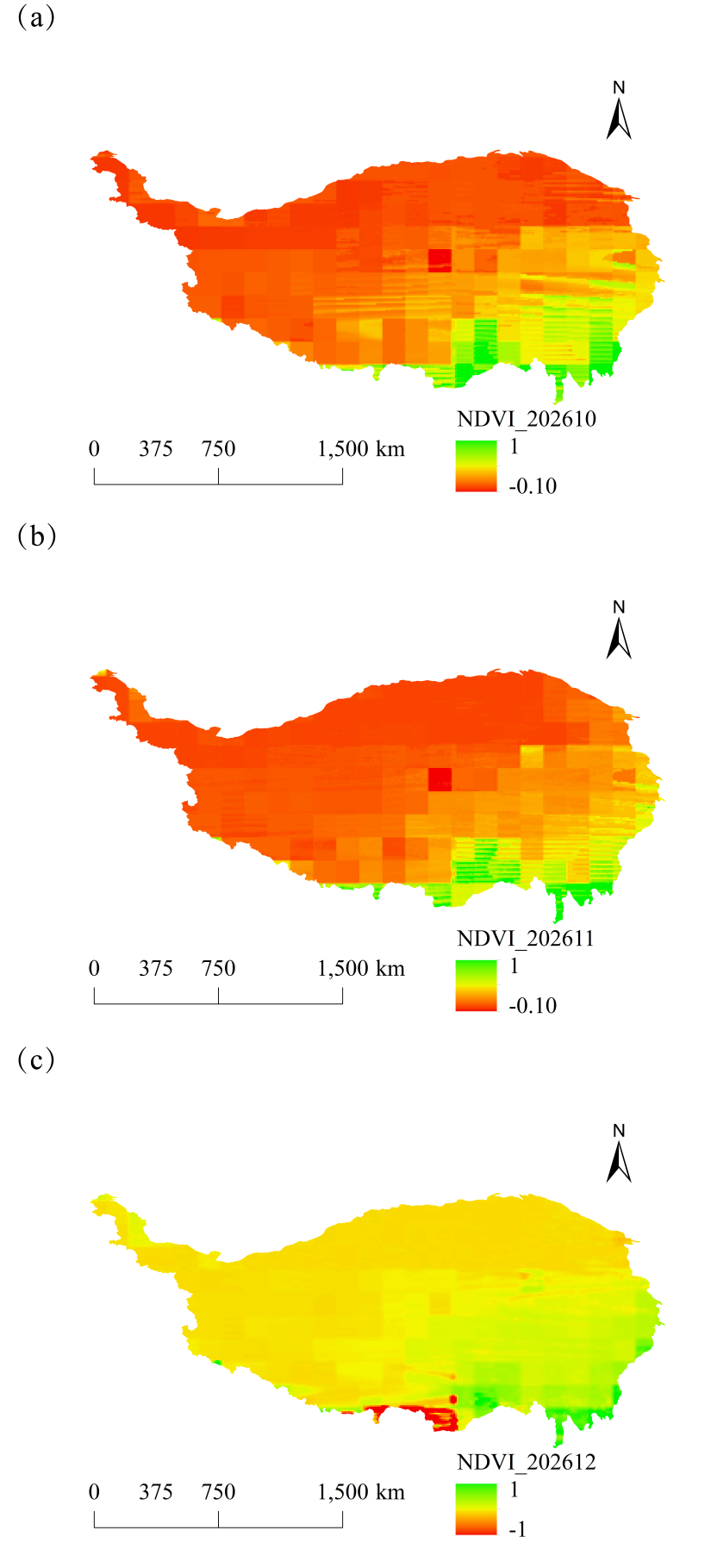
**

**Fig. 9.** Monthly spatial variation of NDVI on the Tibetan Plateau, China, in 2026. (a): October, (b): November, (c): December.

**
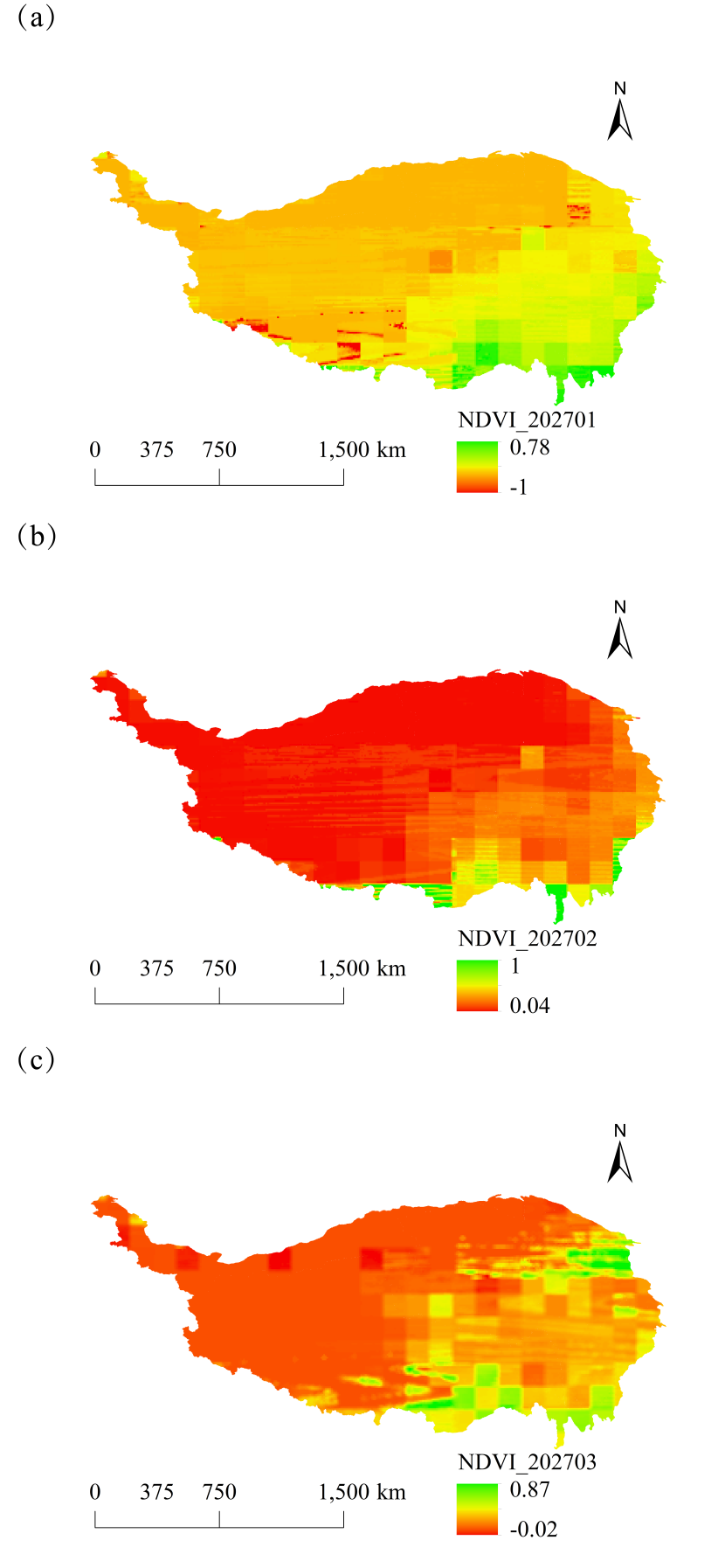
**

**Fig. 10.** Monthly spatial variation of NDVI on the Tibetan Plateau, China, in 2027. (a): January, (b): February, (c): March.

**

Fig. 11.** Monthly spatial variation of NDVI on the Tibetan Plateau, China, in 2027. (a): April, (b): May, (c): June.

**
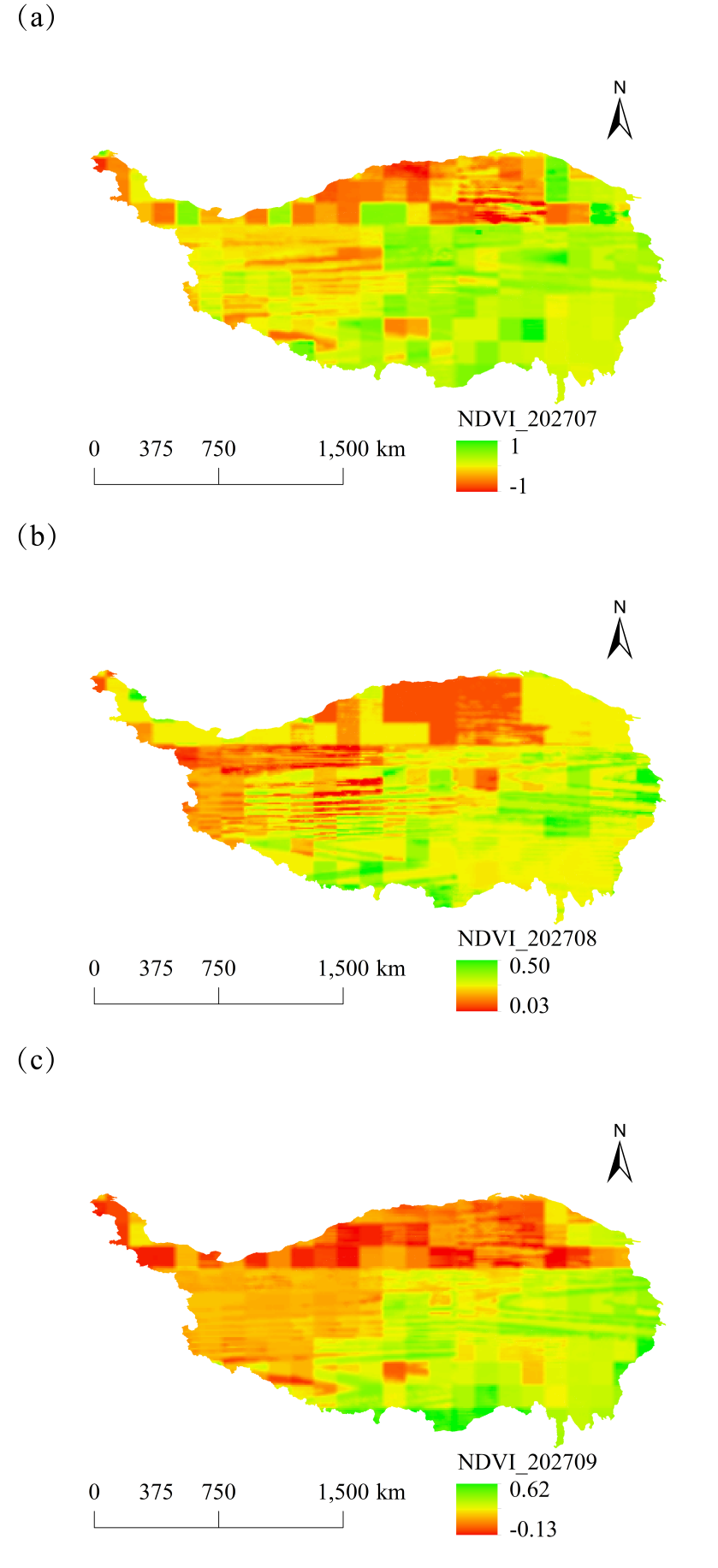
Fig. 12.** Monthly spatial variation of NDVI on the Tibetan Plateau, China, in 2027. (a): July, (b): August, (c): September.


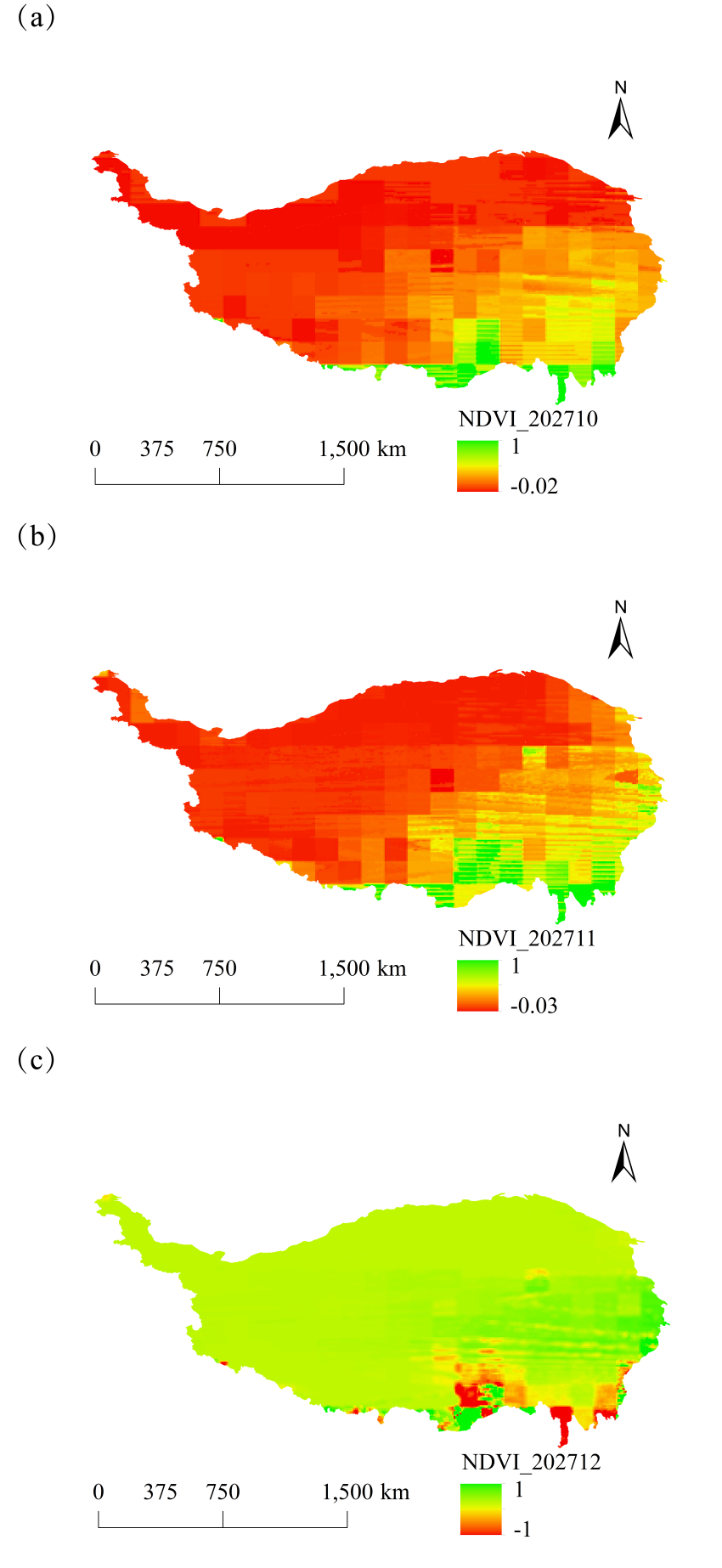


**Fig. 13.** Monthly spatial variation of NDVI on the Tibetan Plateau, China, in 2027. (a): October, (b): November, (c): December.

**
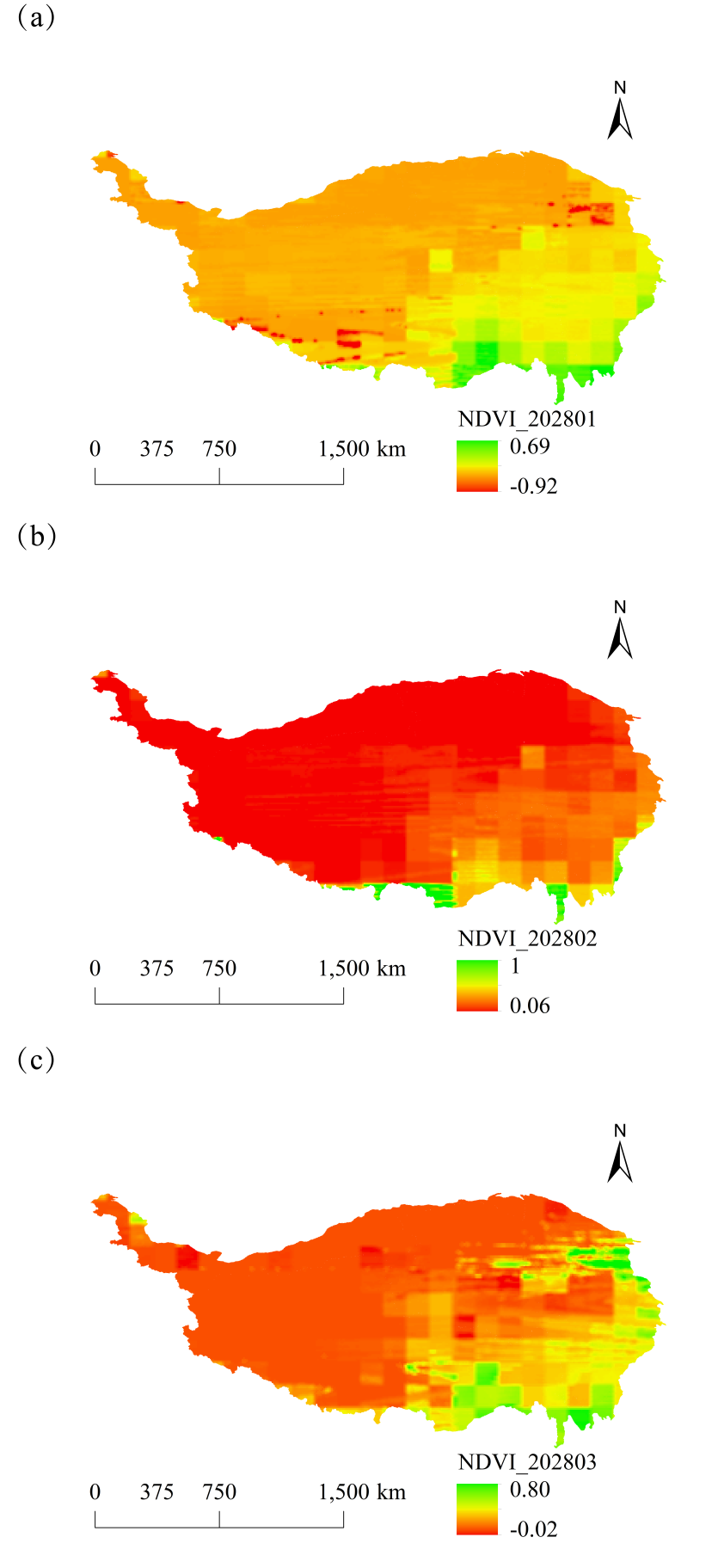
**

**Fig. 14.** Monthly spatial variation of NDVI on the Tibetan Plateau, China, in 2028. (a): January, (b): February, (c): March.





**Fig. 15.** Monthly spatial variation of NDVI on the Tibetan Plateau, China, in 2028. (a): April, (b): May, (c): June.

**
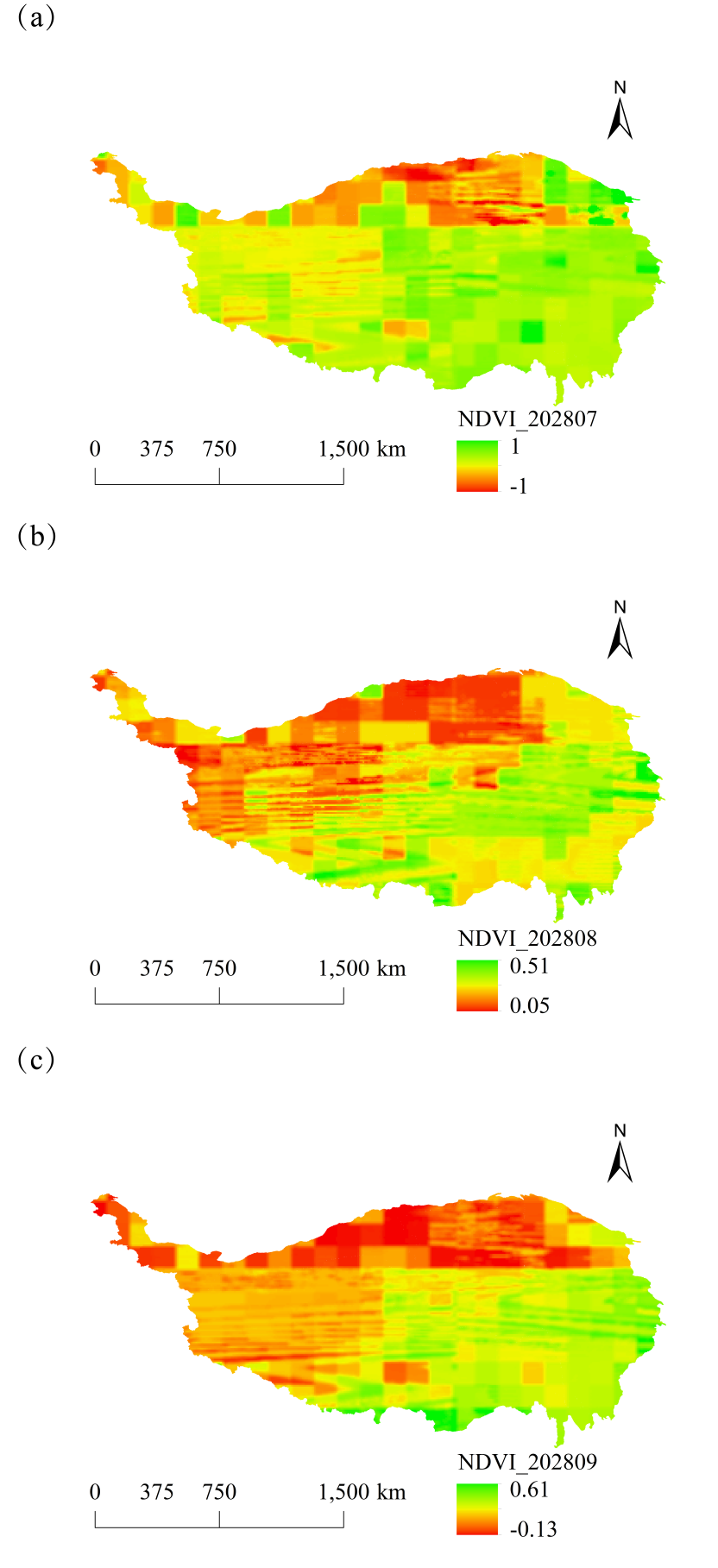
**

**Fig. 16.** Monthly spatial variation of NDVI on the Tibetan Plateau, China, in 2028. (a): July, (b): August, (c): September.


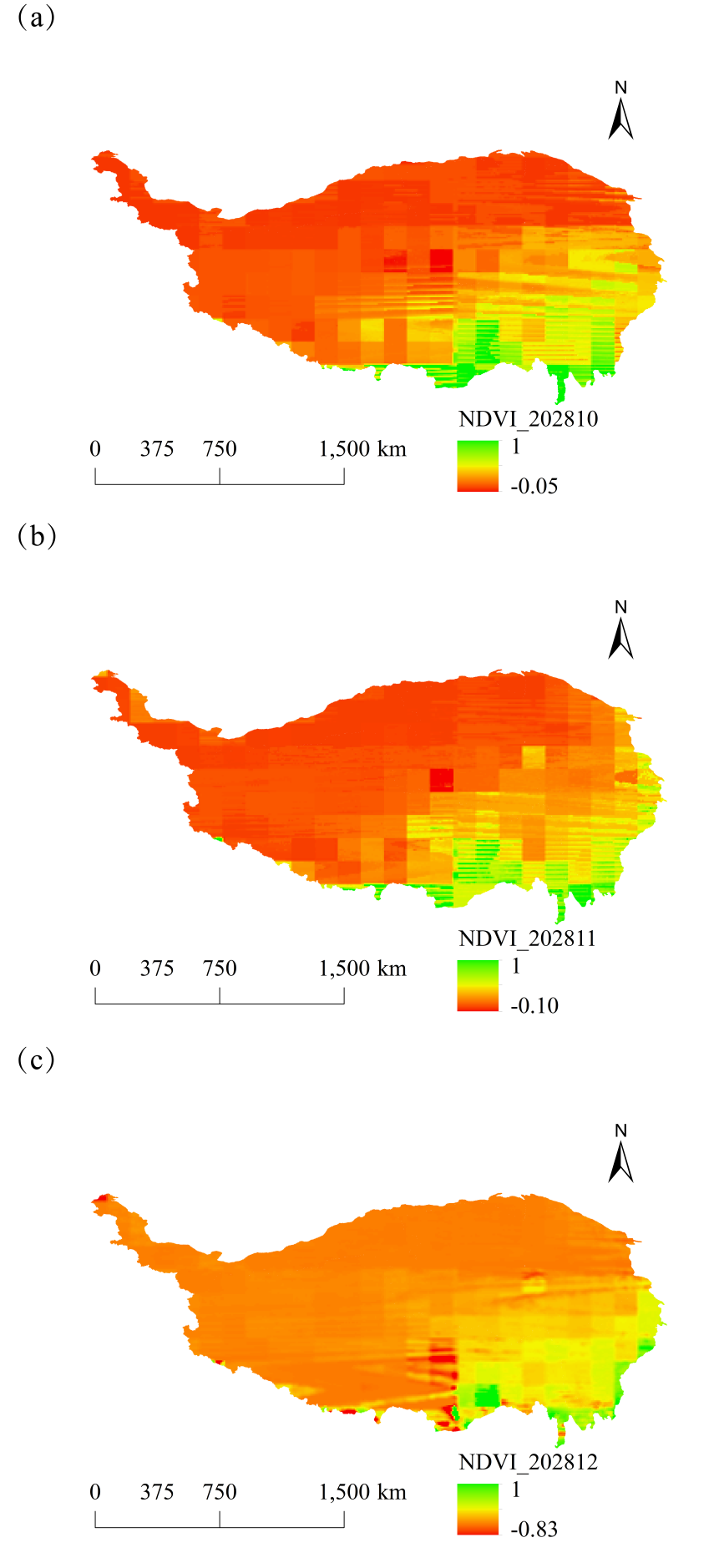
**Fig. 17.** Monthly spatial variation of NDVI on the Tibetan Plateau, China, in 2028. (a): October, (b): November, (c): December.

**
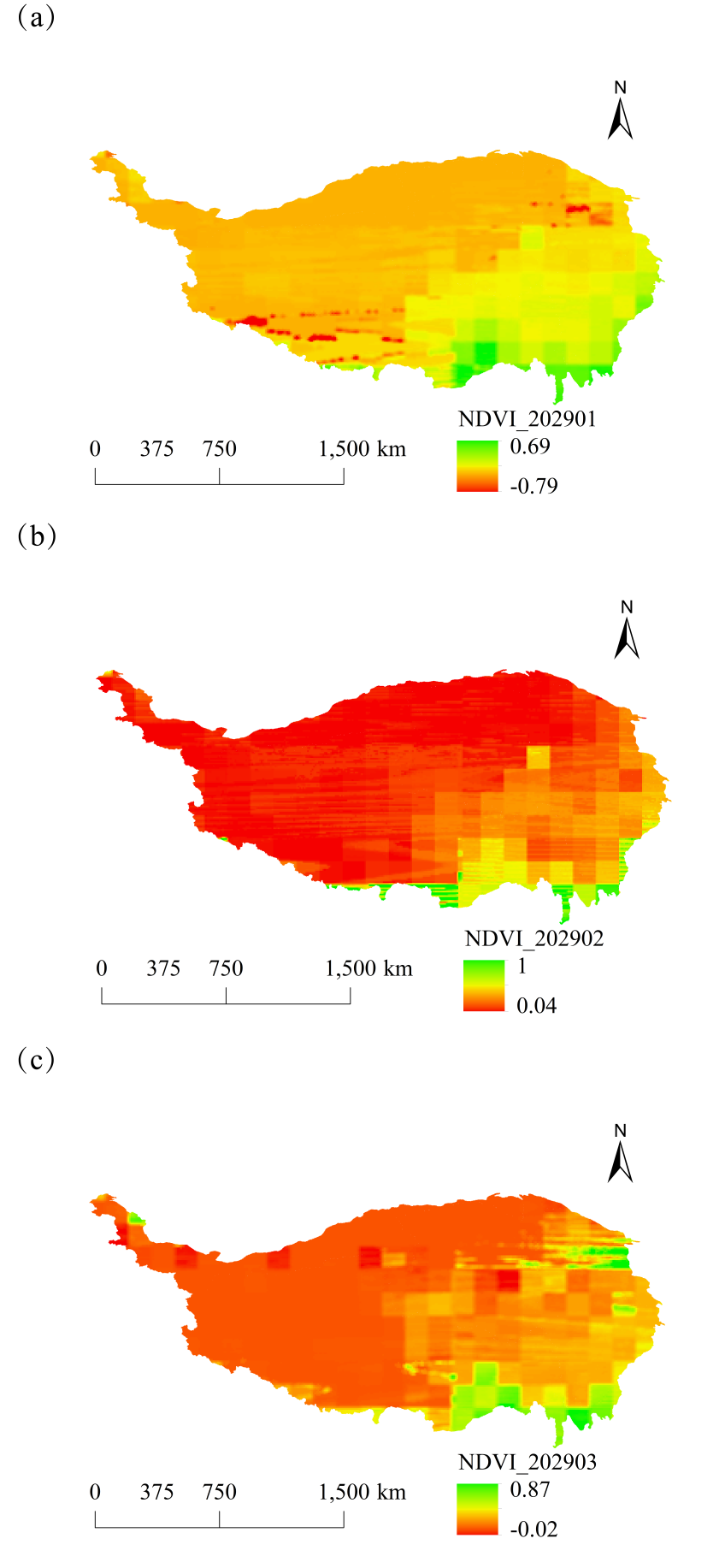
**

**Fig. 18.** Monthly spatial variation of NDVI on the Tibetan Plateau, China, in 2029. (a): January, (b): February, (c): March.


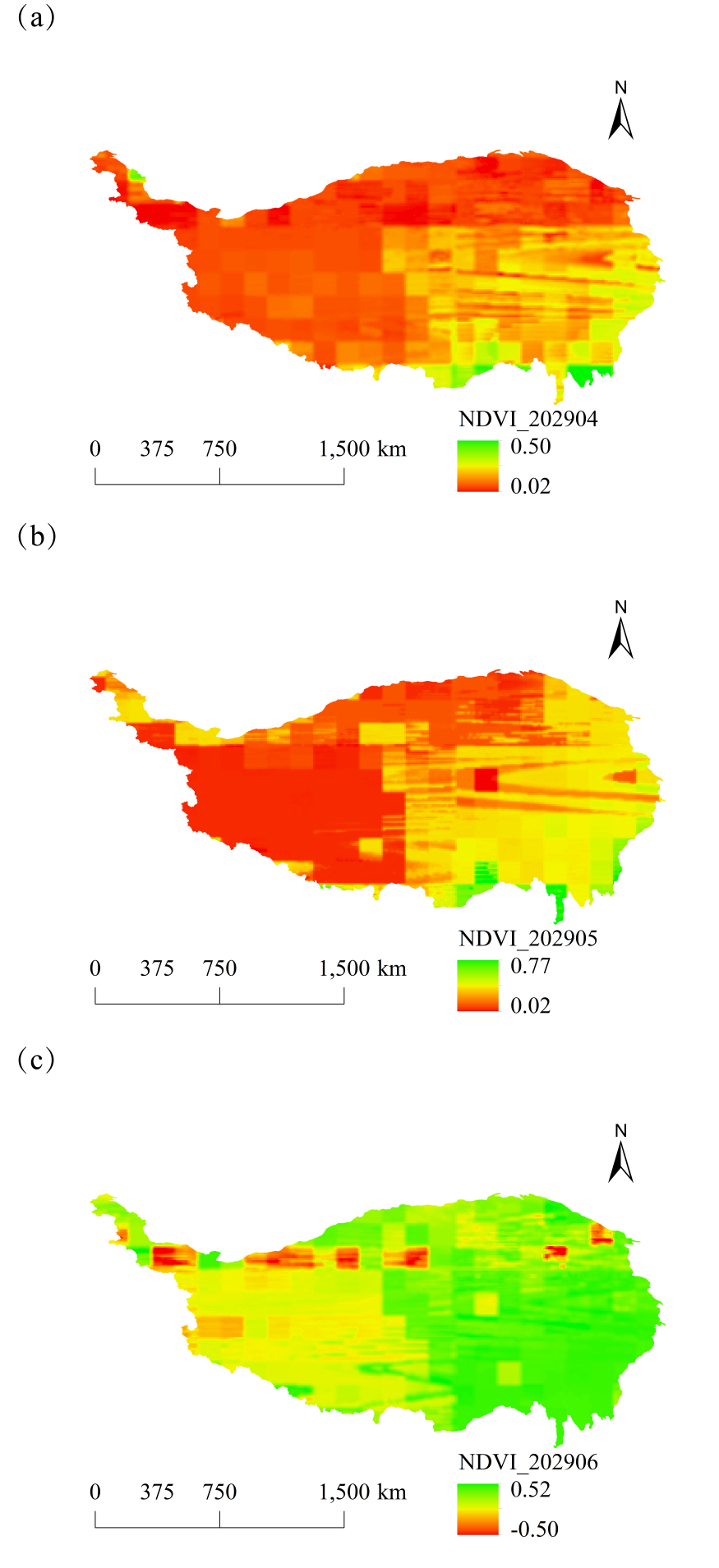
**Fig. 19.** Monthly spatial variation of NDVI on the Tibetan Plateau, China, in 2029. (a): April, (b): May, (c): June.

**
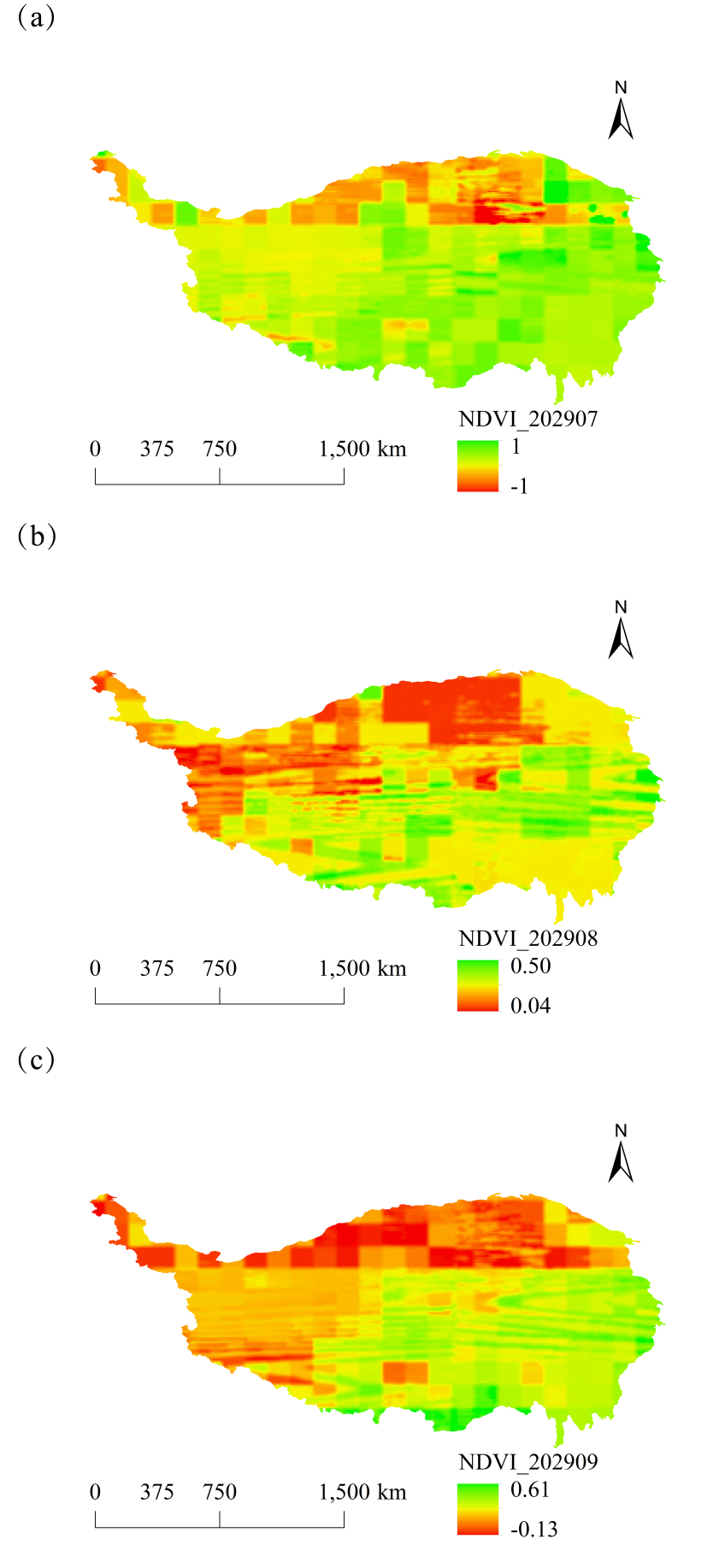
**

**Fig. 20.** Monthly spatial variation of NDVI on the Tibetan Plateau, China, in 2029. (a): July, (b): August, (c): September.

**
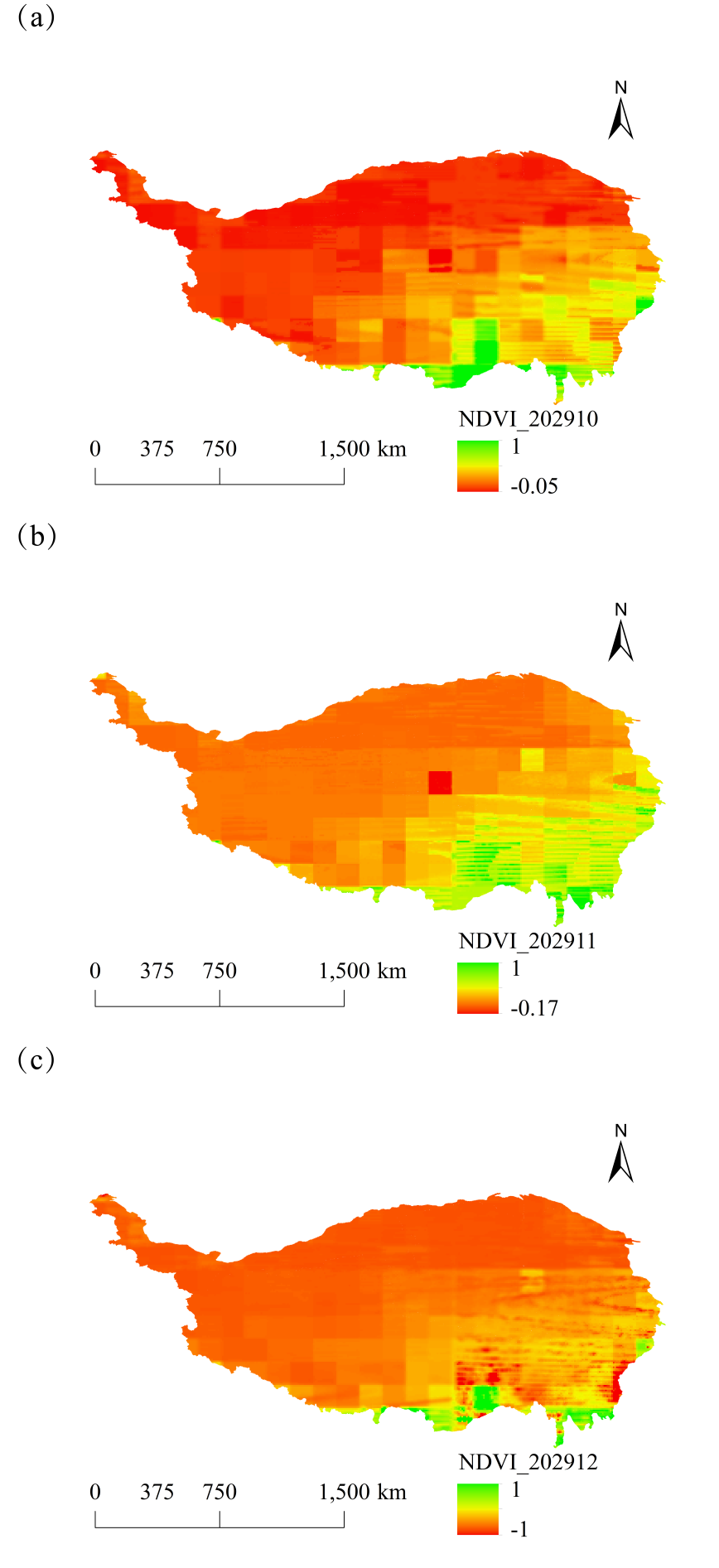
Fig. 21.** Monthly spatial variation of NDVI on the Tibetan Plateau, China, in 2029. (a): October, (b): November, (c): December.


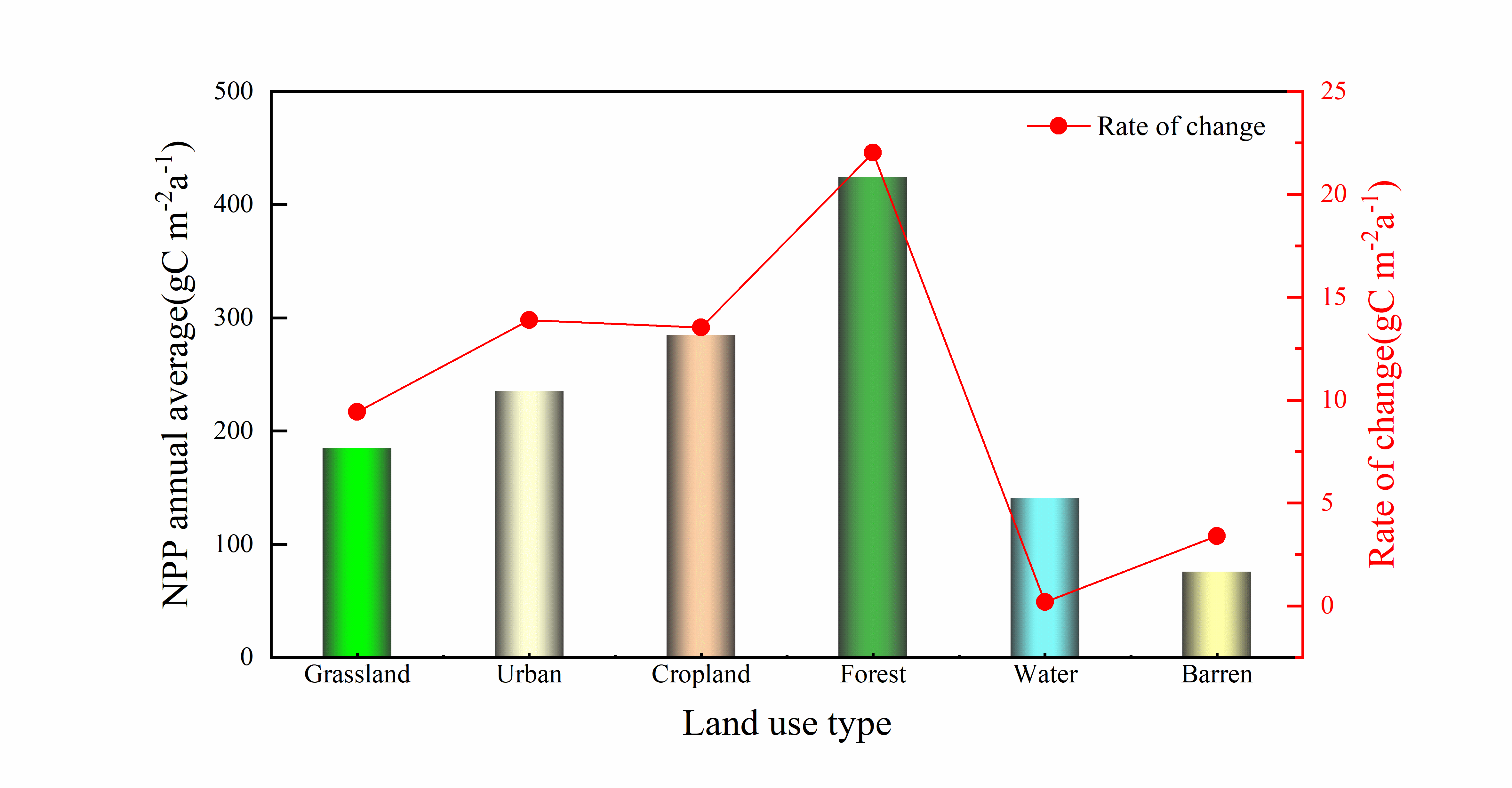


**Fig. 22.** Multi-year averages and rates of change in NPP for different land types, 2025-2030.


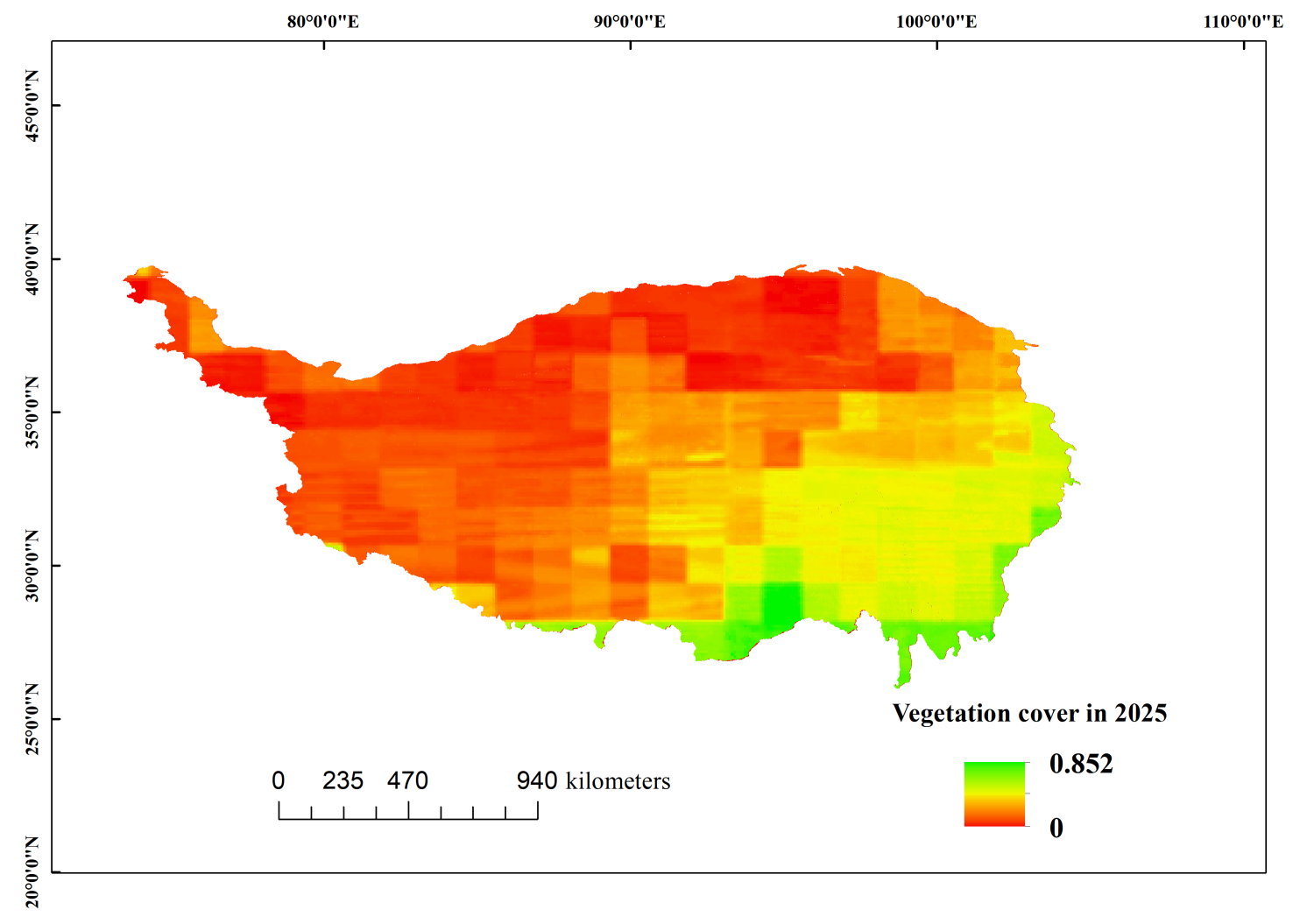


**Fig. 23** Vegetation cover on the Tibetan Plateau, China, in 2025.


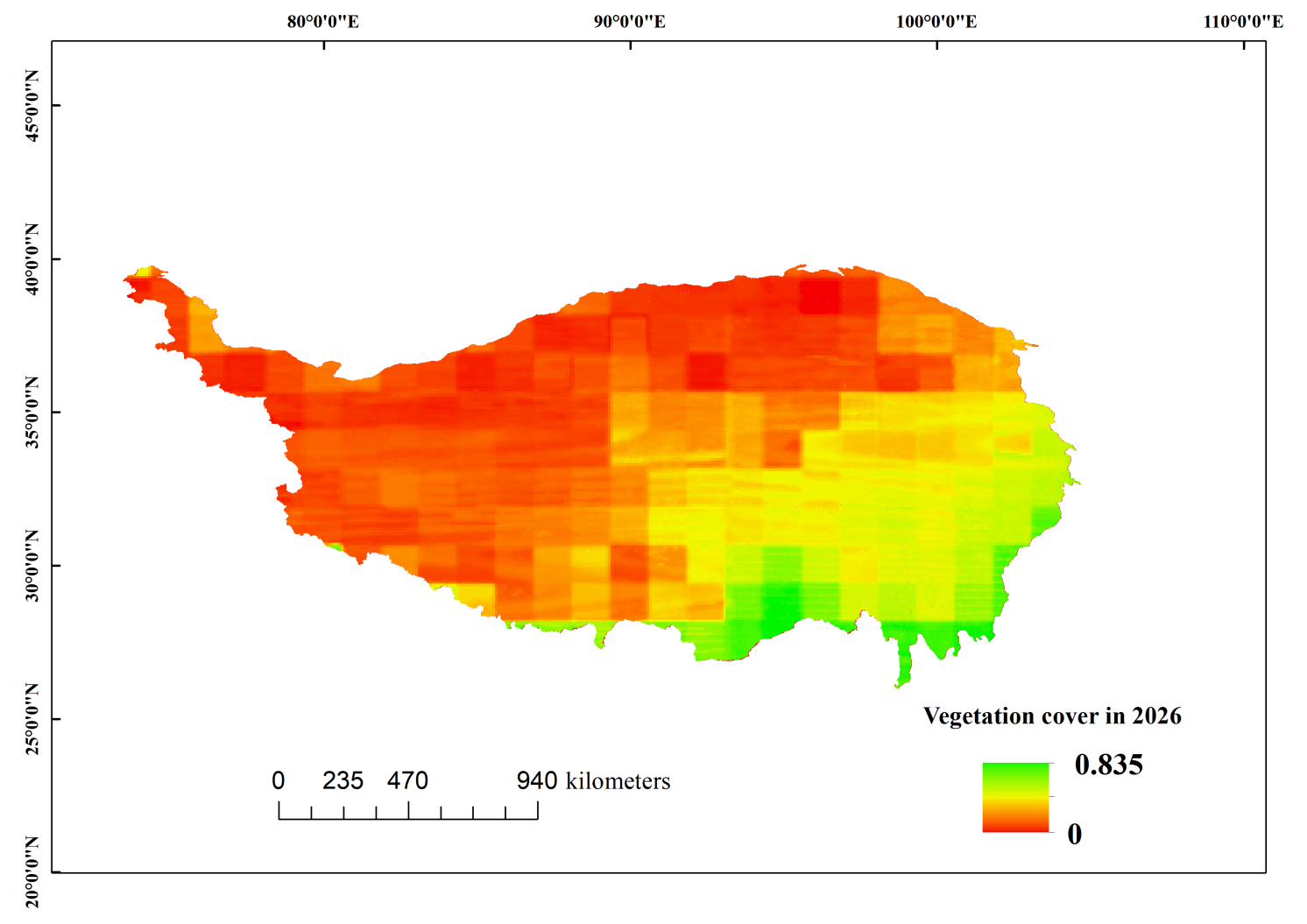
**Fig. 24** Vegetation cover on the Tibetan Plateau, China, in 2026.

**
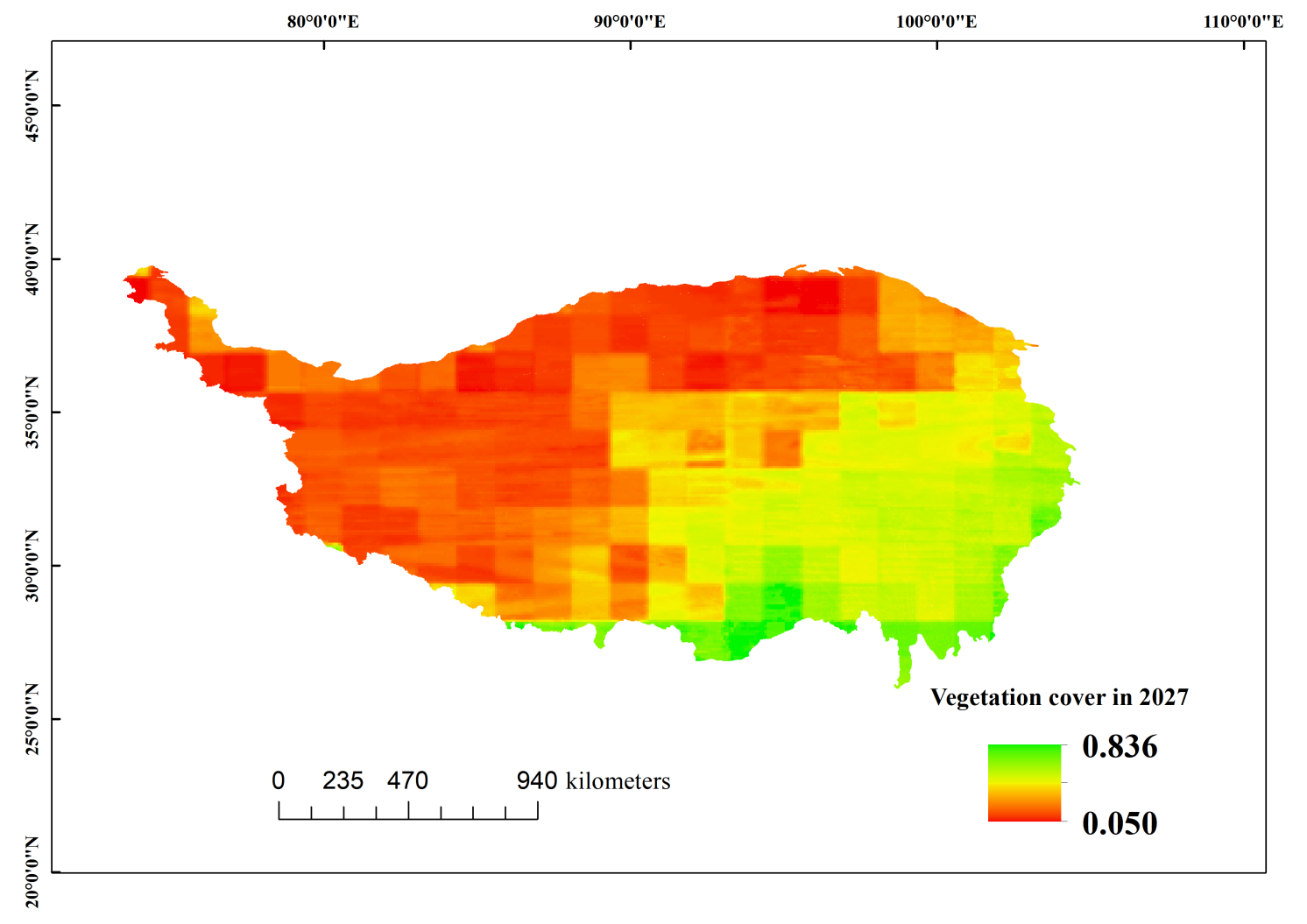
Fig. 25** Vegetation cover on the Tibetan Plateau, China, in 2027.

**
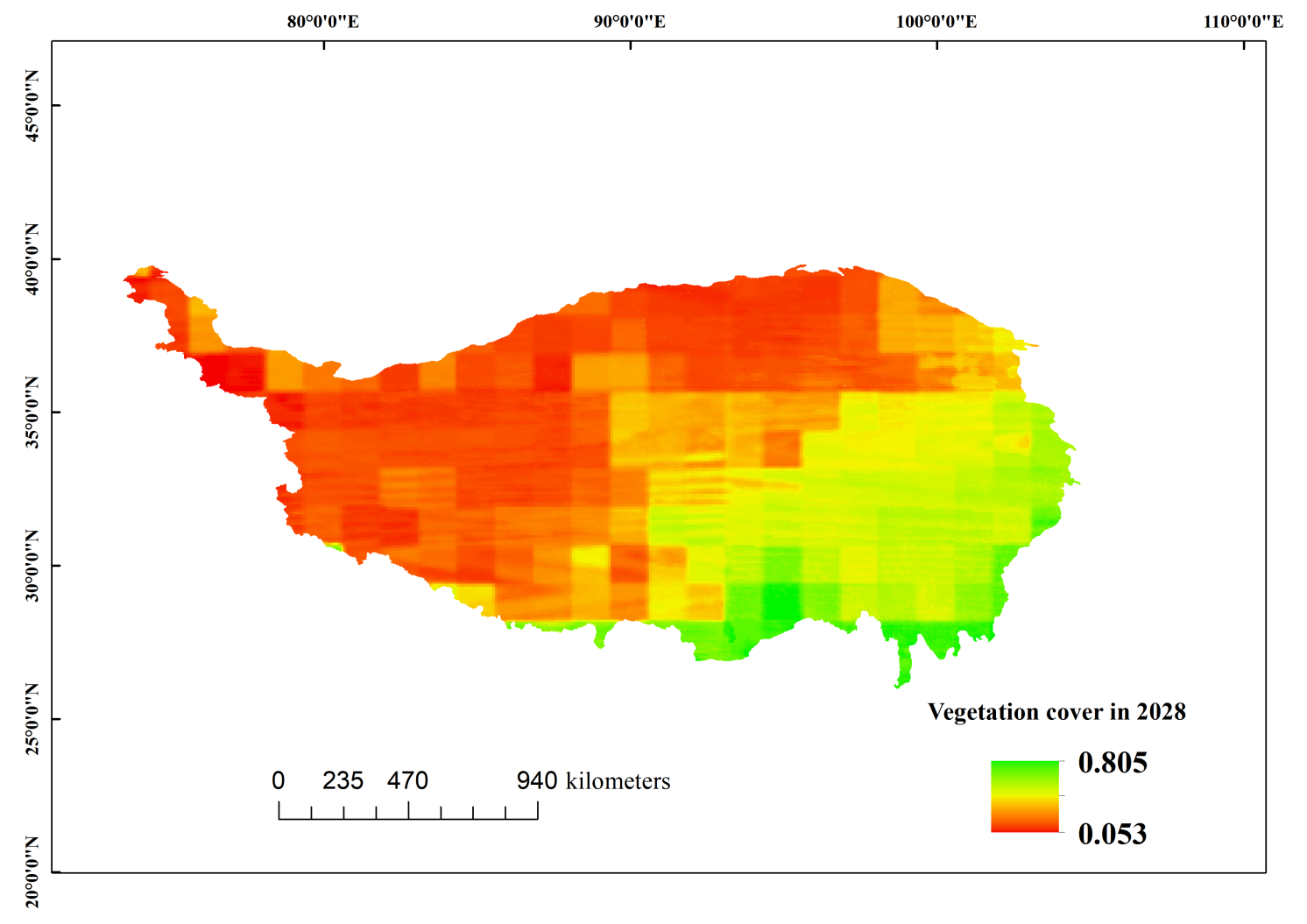
Fig. 26** Vegetation cover on the Tibetan Plateau, China, in 2028.


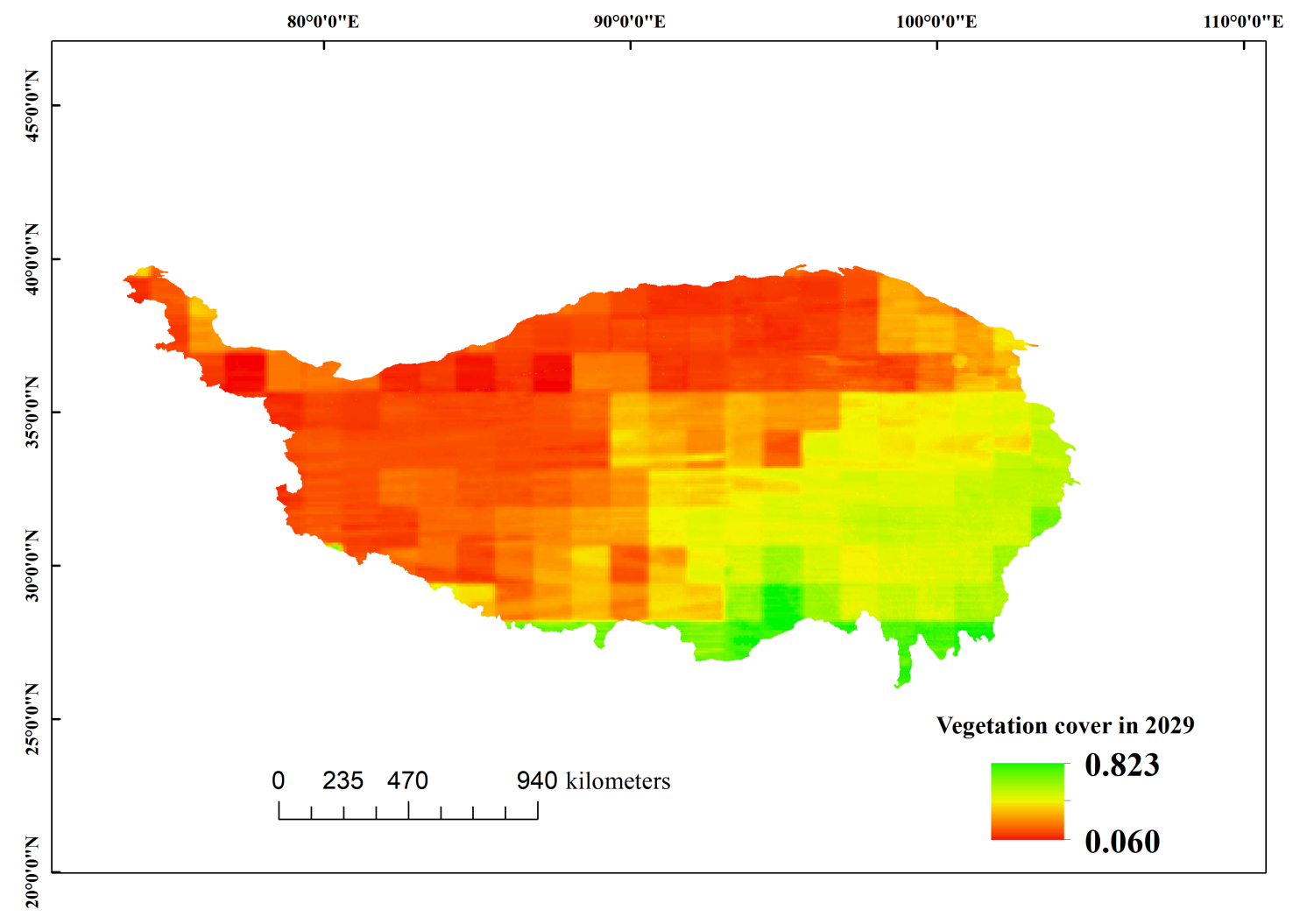
**Fig. 27** Vegetation cover on the Tibetan Plateau, China, in 2029.


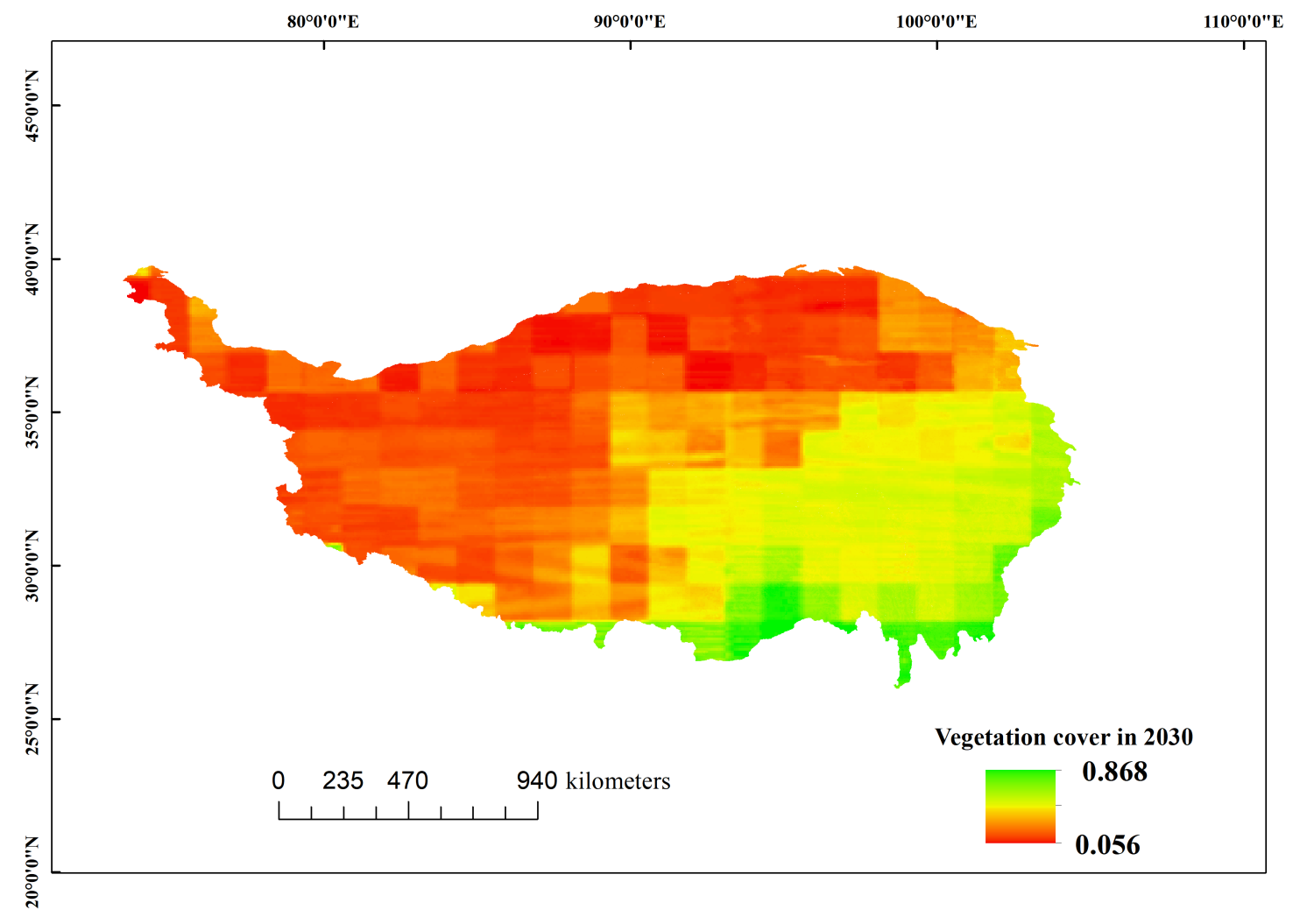
**Fig. 28** Vegetation cover on the Tibetan Plateau, China, in 2030.
